# SIRI+Q model with a limited capacity of isolation

**DOI:** 10.1007/s12064-025-00437-8

**Published:** 2025-03-29

**Authors:** Zhiqiong Fu, Hiromi Seno

**Affiliations:** Department of Computer and Mathematical Sciences, Graduate School of Information Sciences, Tohoku University, Aramaki-Aza-Aoba 6-3-09, Aoba-ku, Sendai, 980-8579 Japan

**Keywords:** Epidemic dynamics, Mathematical model, Isolation, Reinfection, Endemic size, Final epidemic size, 92B99, 92D30, 92D25, 91D99, 00A71

## Abstract

We construct and analyze an SIRI+Q model with a piecewise smooth system of ordinary differential equations for the epidemic dynamics of a reinfectious disease, in which a limited capacity of isolation is incorporated. To consider the relation of the limited isolation capacity to the epidemic consequence, we derive the condition that the isolation reaches the capacity at finite time along the path of the epidemic process, and that the disease becomes endemic. We investigate in particular how the endemicity, the endemic size, or the final epidemic size could depend on the isolation capacity. From the obtained mathematical results, we find theoretical implications on the relevance of the isolation capacity and the difficulty of its measure to control the spread of the disease in the community.

## Introduction

The active globalization of human mobility makes it crucial to consider side effects such as disease spread (Cossar 1994). For history, many infectious diseases disappear, recur, and become less deadly due to people getting immune. Such notable epidemics include “Spanish” flu (1918–1919), Black Deaths (1346–1350) which invaded Europe from Asia and recurred for three decades afterward before getting eliminated (Brauer 2017), SARS beginning with some infection on an airplane in 2003 (Wang and Wu 2018), and the COVID-19 pandemic in this century since mid-December 2019 after the outbreak in China (NIID 2023; CDC 2024; ECDC 2024; WHO 2024). The significantly large number of cases and large-scale spread of the emerging virus about COVID-19 have been initiated and aroused by human mobility at global and local scales (Walters et al. 2018; Du Toit 2020; Liu and Saif 2020; Munster et al. 2020; Phan et al. 2020; Hara and Yamaguchi 2021; Nagata et al. 2021; Ramaswamy et al. 2021; Zhang et al. 2022).

Mathematical modeling of epidemic dynamics could serve to discuss how an infectious disease could spread, the expected duration of the epidemic, the expected number of infected, and the epidemiological indices to characterize the epidemic severity, including the basic reproduction number (Keeling and Rohani 2008; Brauer et al. 2008; Brauer and Castillo-Chavez 2012; Diekmann et al. 2012; Martcheva 2015; Lewis et al. 2019). The early work by Kermack and McKendrick in 1927 is regarded as one of the important origins of mathematical modeling on epidemic dynamics, and has been widely applied for a variety of epidemic problems (Kermack and McKendrick 1927). According to Chowell et al. (2016), it is crucial to formulate reliable models that embody the basic transmission characteristics of specific pathogens and social scenarios. They further stated that improved models are required to capture the variation in early growth dynamics of real epidemics in order to gain a better understanding of the dynamics as they reviewed trends in modeling and classifying early epidemic progression. Recently, mathematical models of the epidemic dynamics are used to estimate or evaluate some epidemiological parameters and to predict the temporal variation in the morbidity about a spreading disease, making use of an epidemiological data (Siettos and Russo 2013). This is particularly the case for the spread of COVID-19 (for example, Kobayashi et al. 2020; Athayde and Alencar 2022; Lin et al. 2022; Musa et al. 2022), while this paper of ours is not the case.

To reduce the risk of the spread of an infectious disease in the community, the strategies of quarantine, isolation, vaccination, and treatment are important. To manage various kinds of infectious diseases like severe acute respiratory syndrome, plague, smallpox, cholera, yellow fever, influenza virus, and SARS-COV-2, the quarantine, isolation, and vaccination are primary. Martcheva (2015) gives a summary of such policies used to manage the spread of infectious diseases. Actually in the pandemic of COVID-19, there have been different policies for the public health from place to place (for example, Pearce et al. 2020; Mendez-Brito et al. 2021; Unruh et al. 2022; Baker et al. 2023). Until now a lot of works have been done with mathematical models including the isolation process for the purpose to consider its contribution to the suppression of a disease spread (for example, Feng and Thieme 1995; Brauer and Castillo-Chavez 2012; Chowell et al. 2016 and references therein). Hethcote et al. (2002) proposed SIR+Q and SIQS models introduced a quarantined/isolated state (Q) with three forms of incidence. In their SIR+Q model with a quarantine-adjusted incidence, the endemic equilibrium is an unstable spiral for a set of parameter values, and a periodic solution arises with Hopf bifurcation. Castillo-Chavez et al. (2003) considered a mathematical model for the purpose to discuss whether the quarantine/isolation can manage the SARS for a limited time frame within a single outbreak. Their model implied that the quarantine/isolation could significantly reduce the size of SARS outbreak. Vivas-Barber et al. (2014) considered an SIR+Q model with the perfect isolation and an asymptomatic state, and got the damped oscillation.

There was a shortage of medical resources in many countries during the COVID-19 outbreak (Unruh et al. 2022). In recent times, some works using mathematical models considered how the limited medical resources could affect the transmission and management of an infectious disease (Abdelrazec et al. 2016; Qin et al. 2016; Wang et al. 2018; Saha and Samanta 2019; Mu et al. 2019; Kumar et al. 2020b; Sepulaveda-Salcedo et al. 2020; Zhao et al. 2020; Wei et al. 2021). Hu et al. (2022) considered an SAIQR model to consider the transmission dynamics of COVID-19 with a limited medical resource under the human migration between two regions, taking account of the asymptomatic state (A). Their results implied that making the basic reproduction number below 1 is not sufficient in order to manage the COVID-19, and it should be significantly below 1. A local outbreak may occur when the medical resources are limited, even if the disease is indexed by a reproduction number below 1.

In addition, the quarantine/isolation may be perfect or imperfect depending on the nature of the epidemic and policies implemented by the community. Erdem et al. (2017) considered a mathematical model for the case of imperfect quarantine/isolation, and found a periodic solution or damped oscillation that indicates recurring outbreaks, depending on the quarantine effectiveness. It is obvious that the isolation requires a specific space with rigorously controlled conditions to keep the infected individuals away from the other community members, so that it must have a certain capacity. With its very small capacity, the isolation strategy may break down at finite time along the path of the epidemic process. Amador and Gomez-Corral (2020) considered a stochastic SIQS model with susceptible, infected, and two quarantine states in which the quarantine has a limited capacity. Their numerical calculation showed a case where the quarantine compartment tends to become full before the outbreak ends, whereas they did not clarify the exact condition for such a case since their numerics were not to discuss the biological meaning of the results but to investigate the mathematical nature of their stochastic model. Since the quarantine/isolation must have an effect on the epidemic dynamics even when it breaks down after a certain moment, we are interested in how the final epidemic/endemic size depends on the isolation capacity.

Ahmad and Seno (2023) considered an SIR+Q model with a system of ordinary differential equations, introducing a limited capacity of isolation. It may be regarded as what is called a *piecewise smooth system*, or sometimes called *Filippov system* or *switching system* (Filippov 1988; Kuznetsov et al. 2003; di Bernardo et al. 2008; di Bernardo and Hogan 2010; Antali and Stepan 2018; Belykh et al. 2023, and references therein). They investigated the dependence of the final epidemic size on the limited isolation capacity, and derived the necessary and sufficient condition that the isolation reaches the capacity at finite time along the path of the epidemic process. The final epidemic size is defined as the proportion of individuals in the community who have experienced the infection until the final stage of the epidemic dynamics. They showed that the final epidemic size could have a discontinuous change at the critical value of isolation capacity below which the isolation reaches the capacity at finite time. Their results imply that the breakdown of isolation with a limited capacity would cause a drastic increase in the final epidemic size. Insufficient capacity of the isolation would lead to an unexpectedly severe epidemic situation, while such a severity could be suppressed with a sufficient isolation capacity.

In this paper, we focus on the relation of such a limited capacity of isolation to the endemicity and the final epidemic/endemic size for a simplest SIRI+Q model on the epidemic dynamics of a reinfectious disease, expanding the modeling by Ahmad and Seno (2023). The reinfectivity of disease in this paper means that the acquired immunity by either vaccination or recovery is imperfect for a reinfectious disease, such that the recovered individuals could have reinfection risk. Actually there are not a few transmissible diseases with a reinfectivity, including influenza (Davies et al. 1984; Hay et al. 2001; Earn et al. 2002; Price et al. 2022; Wang et al. 2022, pertussis (Hethcote 1999; van Boven et al. 2000), Lyme disease (Nadelman et al. 2012), hand, foot, and mouth disease (Zhang et al. 2019), malaria (Arias et al. 2022; Rehman et al. 2022), tuberculosis (Vynnycky and Fine 1997; Horsburgh et al. 2022; Qiu et al. 2022), Ebola virus disease (MacIntyre and Chughtai 2016; Agusto 2017), chronic lung diseases (Yum et al. 2014), invasive pneumococcal disease (Lipsitch 1997), meningococcal disease (Gupta and Maiden 2001), and COVID-19 (Kumar et al. 2020a; Crawford 2022; Le Page 2022; Mensah et al. 2022; Nguyen et al. 2022; Ren et al. 2022; Saad-Roy et al. 2022; Salzer et al. 2022; Shaheen et al. 2022), while the reinfectivity of not a few transmissible diseases has been still requiring scientific researches to understand its kinetics and some other relevant nature.

We will derive the condition that the isolation reaches the capacity at finite time along the path of the epidemic process, and investigate the existence and stability of disease-eliminated and endemic states. Then, we will show that the final epidemic/endemic size would not be necessarily continuous in terms of the isolation capacity, and there is a case where the final epidemic/endemic size depends on the isolation capacity discontinuously at its critical value beyond which the isolation keeps functioning in the epidemic dynamics. Our theoretical results would highlight the importance of satisfactory infrastructure for the public health as indicated by Unruh et al. (2022) on the social response to the COVID-19 pandemic. Since the satisfactory infrastructure for the public health needs a sufficient social investment, arguments on our model would imply a difficulty in the management of even quarantine/isolation policy against an infectious disease spreading in a community too.

## Assumptions

We consider a modeling on the epidemic dynamics of a reinfectious disease during a short-term period, that is, an epidemic season, satisfying the following assumptions on the epidemic dynamics, most of which are the same as those in Ahmad and Seno (2023) except for that about the reinfection:

- The demographic change due to the natural birth, death, and migration is negligible in the season.
- The fatality of disease is negligible in the season.
- The infection occurs by the contact of susceptible individual to not only organic but also potentially inorganic subjects contaminated with the pathogen to cause the disease. This assumption indicates that the considered epidemic dynamics would be on a disease, for example, transmitted with aerosols or droplets emitted from the infective individuals. The transmission may not necessarily require person-to-person contacts.
- The quarantine/isolation/hospitalization has a capacity beyond which the isolation is impossible.
- As long as the isolation has not reached the capacity, the accessibility of the isolation is constant independently of how many infectives are isolated.
- The isolated individuals cannot contact others or be discharged in the epidemic season. Hence, the infectives come to make no contribution to the epidemic dynamics once they enter the isolated state.
- Once the isolation reaches the capacity, its function breaks down to become incapable onward in the season. Then, the epidemic dynamics continues without the quarantine/isolation.
- Even after the recovery from the infection, the individual may get the infection again, that is, the disease is reinfectious.

Since the recovery generates an immunity against the disease, the assumption of possible reinfection means here that the immunity is imperfect or partial against the disease as already mentioned in the introduction section, for example, due to the multiplicity of pathogen types (e.g., mutated variants) (Gökaydin et al. 2007; Wang et al. 2022). As long as we consider a specific pathogen, there may be an immune response as the cross-immunity for the invasion of such similar pathogens by the antigen generated for a specific type of pathogen: The cross-immunity may suppress the reinfection or the effective symptom to reproduce and discharge the pathogen out of the host to cause the disease transmission, while the immunity obtained by the recovery from the disease works only to reduce the risk of reinfection and there is a risk for the recovered individual to get the infection again. For the reasonable modeling, we assume that the reinfection after the recovery from the disease generally has a likelihood not beyond that of the infection for the susceptible.

Since we assume that the reinfection follows the imperfectness of immunity obtained by the recovery from the disease, we will not introduce any specific period or time scale to get reinfected after getting the immunity in our model. Thus, the state transition in terms of the disease follows the susceptible–infective–recovered/immunized–infective (SIRI) structure in our modeling, as used, for example, in Gomes et al. (2004, 2005); Gökaydin et al. (2007); Stollenwerk et al. (2007); Martins et al. (2009); Pinto et al. (2010); Song et al. (2011); Georgescu and Zhang (2013); Guo et al. (2014); Pagliara et al. (2018); Buonomo (2020); Ghosh et al. (2020); Wang (2021); Srivastava and et al. (2022).

## Modeling and model

### Infection and reinfection forces

From the assumption given in the previous section, the reinfection force is introduced here not beyond the infection force $\lambda$ for the susceptible. For the simplest introduction of such a reinfection force, we assume now it as $\varepsilon \lambda$ with a constant $\varepsilon \in [0, 1]$. For the extremal case of $\varepsilon =1$, the recovery does not work at all to reduce the reinfection risk. For $\varepsilon = 0$, the recovery gives the perfect immunity so that there is no likelihood of reinfection. The parameter $\varepsilon$ means the index for the likelihood of reinfection after the recovery.

We introduce the infection force $\lambda$ for the susceptible in this paper as

1 $$\begin{aligned} \lambda =\lambda (I, Q) := \beta \dfrac{I}{N-Q}, \end{aligned}$$

where *I* and *Q* are, respectively, the infective and isolated population sizes (densities), *N* total population size in the community, and $\beta$ the infection coefficient. This formula of the infection force is lead from the following idea with the assumption on the transmission route through the subjects contaminated with the pathogen. For the continuous time model, the infection force is generally defined by the probability of infection per susceptible individual in a sufficiently short time interval $\varDelta t$, which is mathematically given as $\lambda \varDelta t +\mathrm o(\varDelta t)$.

We ignore any change/shift in the custom and style of daily life in the community under the epidemic dynamics. This indicates an assumption that the free (non-isolated) individual has a daily life independent of the situation of epidemic dynamics. Then, the free individual is assumed to have a probability to contact to the subjects which may be contaminated with the pathogen, given by $c\varDelta t +\mathrm o(\varDelta t)$ with a positive constant *c* in a sufficiently short time interval $\varDelta t$. The frequency of such contacts to the subjects depends only on the custom and style of daily life, and it is now assumed to be represented by a constant *c*. The probability of the contact to contagious subjects is assumed to be proportional to the ratio of infective population density *I* to the free population density $N-Q$, that is given by $I/(N-Q)$. In other words, with the mean-field approximation, the probability that a subject is contaminated by the pathogen is assumed to be proportional to $I/(N-Q)$. The infection by such a contagious contact follows a probability characterizing the infectivity of the pathogen too. The product of these three factors results in the infection probability per susceptible individual in a sufficiently short time interval $\varDelta t$, given as $\lambda (I, Q)\varDelta t+\mathrm o(\varDelta t)$ by the above formula (1) with the infection coefficient $\beta$ representing the constant parameters determined by those three factors.

### Epidemic phases

From the assumptions of the availability of isolation, we need to take account of two different *epidemic phases* in our modeling, as Ahmad and Seno (2023) did: *isolation effective phase* and *isolation incapable phase*.

*Isolation effective phase:* This is the epidemic phase at which the isolated subpopulation size *Q* is less than the capacity, a given positive constant $Q_{\textrm{max}}$, when the isolation works with quarantine/isolation rate $\sigma$. The epidemic dynamics at this phase is governed by

2 $$\begin{aligned} \begin{aligned} \dfrac{\hbox {d}S}{\hbox {d}t}&=-\beta \dfrac{I}{N-Q}S;\\ \dfrac{\hbox {d}I}{\hbox {d}t}&= \beta \dfrac{I}{N-Q}S+\varepsilon \beta \dfrac{I}{N-Q}R-\rho I-\sigma I;\\ \dfrac{\hbox {d}Q}{\hbox {d}t}&=\sigma I;\\ \dfrac{\hbox {d}R}{\hbox {d}t}&=\rho I-\varepsilon \beta \dfrac{I}{N-Q}R. \end{aligned} \end{aligned}$$

The variables *S*, *I*, and *R* denote the sizes of susceptible, infective, and recovered subpopulations, respectively. The total population size of the community is denoted by a positive constant *N*, and it is satisfied that $S(t)+I(t)+Q(t)+R(t)=N$ for any $t\ge 0$. The parameter $\rho$ denotes the natural recovery rate of infective individual. The reinfection coefficient is given by $\varepsilon \beta$, where $0<\varepsilon <1$, as given in the previous section. The quarantine/isolation rate of infective individual at this phase $\sigma$ represents the efficiency of quarantine operation to detect and isolate an infective.

*Isolation incapable phase:* This is the epidemic phase at which the isolated subpopulation size *Q* has reached the capacity $Q_{\textrm{max}}$, and then the isolation breaks down to become incapable. The epidemic dynamics at this phase is governed by

3 $$\begin{aligned} \begin{aligned} \dfrac{\hbox {d}S}{\hbox {d}t}&=-\beta \dfrac{I}{N-Q_{\textrm{max}}}S;\\ \dfrac{\hbox {d}I}{\hbox {d}t}&= \beta \dfrac{I}{N-Q_{\textrm{max}}}S+\varepsilon \beta \dfrac{I}{N-Q_{\textrm{max}}}R-\rho I;\\ \dfrac{\hbox {d}Q}{\hbox {d}t}&=0;\\ \dfrac{\hbox {d}R}{\hbox {d}t}&=\rho I-\varepsilon \beta \dfrac{I}{N-Q_{\textrm{max}}}R. \end{aligned} \end{aligned}$$

Once the isolation reaches the capacity, the system switches to the isolation incapable phase. Since we assume no discharge of isolated infectives from the isolation state, the subpopulation size of free individuals is $N-Q_{\textrm{max}}$ at this phase. The extremal case with $Q_{\textrm{max}}\ge N$ corresponds to the situation where the isolation never reaches the capacity, that is, it always works in the epidemic dynamics. Only if $Q_{\textrm{max}}< N$, the isolation could reach the capacity to cease functioning. Therefore, we consider hereafter only the case of $Q_{\textrm{max}}< N$ as a reasonable setup for our model in this paper.

### Full system for epidemic dynamics

**Fig. 1:**
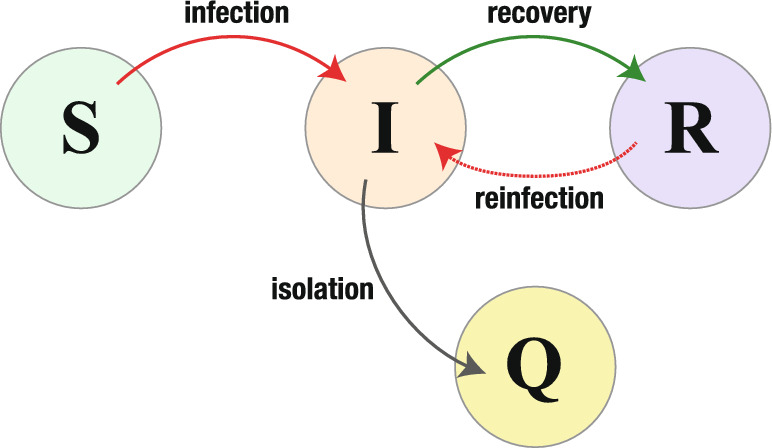
The individual state transition according to the epidemic dynamics of our model (4)

With the above modeling of the epidemic dynamics at two different epidemic phases, we shall consider the following system as our mathematical model in this paper:

4 $$\begin{aligned} \begin{aligned} \dfrac{\hbox {d}S}{\hbox {d}t}&=-\beta \dfrac{I}{N-Q}S;\\ \dfrac{\hbox {d}I}{\hbox {d}t}&= \beta \dfrac{I}{N-Q}S+\varepsilon \beta \dfrac{I}{N-Q}R-\rho I-\Phi (Q, I);\\ \dfrac{\hbox {d}Q}{\hbox {d}t}&=\Phi (Q, I);\\ \dfrac{\hbox {d}R}{\hbox {d}t}&=\rho I-\varepsilon \beta \dfrac{I}{N-Q}R \end{aligned} \end{aligned}$$

with

$$\begin{aligned} \Phi (Q, I) = \left\{ \begin{array}{cc} \sigma I & \hspace{4mm} \text{ for } Q< Q_{\textrm{max}}; \\ 0 & \hspace{4mm} \text{ for } Q=Q_{\textrm{max}}, \\ \end{array} \right. \end{aligned}$$

and the initial condition $(S(0), I(0), Q(0), R(0)) = (S_0, I_0, 0, 0)$ where $S_0> 0$, $I_0> 0$, and $S_0+I_0 = N$. The individual state transition according to the epidemic dynamics is schematically shown in Fig. 1. This model with $\varepsilon = 0$ coincides with the SIR+Q model in Ahmad and Seno (2023).

The piecewise function $\Phi (Q,I)$ denotes the net quarantine/isolation rate of infected individuals. As long as the isolated subpopulation size *Q* is less than the capacity $Q_{\textrm{max}}$, the isolation is available, and the epidemic dynamics is at the isolation effective phase with $\Phi (Q,I)=\sigma I$. Once *Q* reaches $Q_{\textrm{max}}$, the isolation becomes ceased after it. Then, the epidemic dynamics switches to the isolation incapable phase with $\Phi (Q,I)=0$.

As was the model considered by Ahmad and Seno (2023), our model (4) may be regarded as a *piecewise smooth system*, especially what is sometimes called *Filippov system* or *switching system* (Filippov 1988; Kuznetsov et al. 2003; di Bernardo et al. 2008; di Bernardo and Hogan 2010; Antali and Stepan 2018; Belykh et al. 2023, and references therein), while we shall not intend to analyze the system (4) deeply as such a Filippov system in applied mathematics since we focus on the discussion about the relation of the isolation capacity to the endemicity and the final epidemic/endemic size for the SIRI+Q model (4) on the epidemic dynamics of a reinfectious disease.

With the transformation of variables and parameters,

$$\begin{aligned} \begin{aligned}&\tau :=(\rho +\sigma ) t;\quad u :=\dfrac{\, S\, }{N};\quad v :=\dfrac{\, I\, }{N};\quad q :=\dfrac{\, Q\, }{N};\quad w :=\dfrac{\, R\, }{N}; \\&\gamma :=\dfrac{\,\sigma \,}{\rho +\sigma };\quad q_{\textrm{max}}:=\dfrac{\ Q_{\textrm{max}}}{N};\quad {\mathscr {R}}_0 :=\dfrac{\,\beta \,}{\rho +\sigma }, \end{aligned} \end{aligned}$$

and with the basic reproduction number ${\mathscr {R}}_0:=\beta / (\rho +\sigma )$ for the model (4), we can derive the following non-dimensionalized system mathematically equivalent to the system (4):

5 $$\begin{aligned} \begin{aligned} \dfrac{\hbox {d}u }{\hbox {d}\tau }&= -{\mathscr {R}}_0\dfrac{ v }{1- q } u; \\ \dfrac{\hbox {d}v }{\hbox {d}\tau }&= {\mathscr {R}}_0\dfrac{ v }{1- q } u +\varepsilon {\mathscr {R}}_0\dfrac{ v }{1- q } w - (1-\gamma )v -\phi (q, v); \\ \dfrac{\hbox {d}q }{\hbox {d}\tau }&= \phi (q, v); \\ \dfrac{\hbox {d}w }{\hbox {d}\tau }&= (1-\gamma )v -\varepsilon {\mathscr {R}}_0\dfrac{ v }{1- q } w \end{aligned} \end{aligned}$$

with

$$\begin{aligned} \phi (q, v) = \left\{ \begin{array}{cc} \gamma v& \hspace{4mm} \text{ for } q < q_{\textrm{max}}; \\ 0& \hspace{4mm} \text{ for } q = q_{\textrm{max}}, \end{array} \right. \end{aligned}$$

and the initial condition $( u (0), v (0), q (0), w (0)) = ( u _0, v _0, 0, 0)$ where $u_0> 0$ and $v_0 = 1-u_0> 0$. We will consider only the case of $q_{\textrm{max}} < 1$ as already mentioned in the previous section. For a mathematical convention, we show here the following mathematical feature about the solution of (5) (Appendix A):

#### Lemma 3.1

For the initial condition $(u(0), v(0), q(0), w(0)) = (u_0, v_0, 0, 0)$ with $v_0>0$ and $u_0 = 1-v_0> 0$, the solution of (5) belongs to the set $\{ (u, v, q, w)\in {\mathbb {R}}^4_+\mid u+v+q+w = 1 \}$ for $\tau> 0$.

**Fig. 2:**
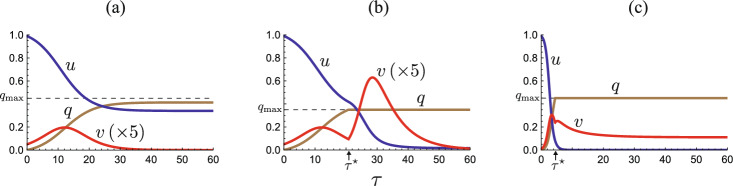
Numerical examples for the temporal variation of the model (5). **a** $q_{\textrm{max}}=0.45$, ${\mathscr {R}}_0 = 1.2$ ($\varepsilon {\mathscr {R}}_0 = 0.24$); **b** $q_{\textrm{max}}=0.35$, ${\mathscr {R}}_0 = 1.2$ ($\varepsilon {\mathscr {R}}_0 = 0.24$); **c** $q_{\textrm{max}}=0.45$, ${\mathscr {R}}_0 = 2.5$ ($\varepsilon {\mathscr {R}}_0 = 0.50$). Commonly, $u_0=0.99$; $\varepsilon = 0.2$; $\gamma = 0.6$. In (**a**), the isolation never reaches the capacity, while it reaches the capacity and becomes incapable after a moment $\tau = \tau ^\star$ in (**b**) and (**c**)

As numerically exemplified by Fig. 2a for a sufficiently large capacity $q_{\textrm{max}}$, the epidemic dynamics can always remain at the isolation effective phase with $\phi (q, v)=\gamma v$, when the isolation never reaches the capacity. In contrast, as numerically exemplified by Fig. 2b, c, if the isolation capacity is insufficient, it reaches the capacity, and then the isolated subpopulation size *q* remains $q_{\textrm{max}}$ at the isolation incapable phase since any isolated individual is not discharged from the isolation, following the assumption and modeling given in the previous and present sections.

## Conserved quantities in the epidemic dynamics

In addition to the time-independent equality $u+v+q+w = 1$, we can find the following time-independent equalities for the variables as the conserved quantities in the epidemic dynamics governed by the system (5) (Appendix B).

*At the isolation effective phase:*

6 $$\begin{aligned} 1-q&=\Big (\dfrac{u}{\, u_0}\Big )^{\gamma / {\mathscr {R}}_0}; \end{aligned}$$

7 $$\begin{aligned} u+v&=F(u):= \left\{ \begin{array}{ll} \dfrac{1-\varepsilon {\mathscr {R}}_0 }{\gamma -\varepsilon {\mathscr {R}}_0} \Big (\dfrac{u}{\, u_0}\Big )^{\gamma / {\mathscr {R}}_0} -\dfrac{1-\gamma }{\gamma -\varepsilon {\mathscr {R}}_0} \Big (\dfrac{u}{\, u_0}\Big )^{\varepsilon } & \text{ for }\ \varepsilon {\mathscr {R}}_0\ne \gamma ; \\ \Big ( 1 + \varepsilon \dfrac{1-\gamma }{\gamma } \ln \dfrac{u}{\, u_0} \Big ) \Big (\dfrac{u}{\, u_0}\Big )^{\varepsilon } & \text{ for }\ \varepsilon {\mathscr {R}}_0= \gamma . \end{array} \right. \end{aligned}$$

* At the isolation incapable phase:*

8 $$\begin{aligned} &q= q_{\textrm{max}};\\ &u+v=G(u) := \Big (1-\dfrac{1-\gamma }{\varepsilon {\mathscr {R}}_0}\Big )(1-q_{\textrm{max}})+B\Big (\dfrac{u}{\, u_0}\Big )^{\varepsilon } \end{aligned}$$

with

9 $$\begin{aligned} B:= \left\{ \begin{array}{ll} \dfrac{1-\gamma }{\varepsilon {\mathscr {R}}_0(1-\varepsilon {\mathscr {R}}_0/\gamma )} \Big [(1-q_{\textrm{max}})^{1-\varepsilon {\mathscr {R}}_0/\gamma }-\dfrac{\varepsilon {\mathscr {R}}_0}{\gamma }\Big ] & \text{ for }\ \varepsilon {\mathscr {R}}_0\ne \gamma ; \\ \dfrac{1-\gamma }{\gamma } \big [ \ln (1-q_{\textrm{max}})+1 \big ] & \text{ for }\ \varepsilon {\mathscr {R}}_0= \gamma . \end{array} \right. \end{aligned}$$

The equations for $\varepsilon {\mathscr {R}}_0= \gamma$ in (7) and (9) can be mathematically derived also by taking the limit as $\varepsilon {\mathscr {R}}_0\rightarrow \gamma$ for those for $\varepsilon {\mathscr {R}}_0\ne \gamma$. Hence, we may use only the equations for $\varepsilon {\mathscr {R}}_0\ne \gamma$ without distinguishing the case of $\varepsilon {\mathscr {R}}_0= \gamma$ unless it would be necessary in the mathematical argument. That is, we may use the equations for $\varepsilon {\mathscr {R}}_0\ne \gamma$ as those mathematically including the specific case of $\varepsilon {\mathscr {R}}_0= \gamma$.

As described about the derivation of (8) in Appendix B, we used the continuity of the temporal variation of the variables in the system (5) at the moment that the isolation reaches the capacity and the system (5) switches to the isolation incapable phase. Then, we have noted the following feature of the system (5), which will be useful for our subsequent mathematical analysis on the model:

### Lemma 4.1

If the system enters the isolation incapable phase at a finite time $\tau =\tau ^\star$, then the susceptible subpopulation size at the moment becomes

10 $$\begin{aligned} u(\tau ^\star ) = u^\star := u_0\big (1-q_{\textrm{max}}\big )^{{\mathscr {R}}_0/\gamma }. \end{aligned}$$

Note that, from the continuity of variables *u* and *v* at $\tau =\tau ^\star$, equalities (6), (7), and (8) simultaneously holds at $\tau =\tau ^\star$, so that we have $F(u^\star ) = G(u^\star )$.

## Equilibrium for the isolation effective phase

In this section, suppose that the system (5) always remains at the isolation effective phase, when it never reaches its capacity at finite time along the path of the epidemic process. Then with the arguments given in Appendix C, we can obtain the following results on the consequence of the epidemic dynamics when the system (5) always remains at the isolation effective phase.

First, we find the following result implying that a sufficiently large isolation capacity could lead the system to a disease-eliminated equilibrium $E_0^{-}$, even though the disease is reinfectious for the recovered individuals (Appendix C):

### Lemma 5.1

If the system always remains at the isolation effective phase, the disease is eventually eliminated.

Next, we can obtain the following important feature of the epidemic dynamics by the system (5):

### Lemma 5.2

If the system (5) can always remain at the isolation effective phase, there are necessarily some susceptibles who can escape from the infection at the end of the epidemic dynamics.

The existence of such susceptibles at the end of the epidemic dynamics is well known for the Kermack-McKendrick SIR model (Brauer et al. 2008; Keeling and Rohani 2008; Brauer and Castillo-Chavez 2012; Diekmann et al. 2012; Martcheva 2015; Seno 2022), while the above lemma indicates such a case even for the epidemic dynamics with a reinfectious disease in our model.

Consequently with these lemmas, we can obtain the following result (Appendix C):

### Theorem 5.1

If the system always remains at the isolation effective phase, it eventually approaches a disease-eliminated equilibrium $E_0^{-}$ given by

11 $$\begin{aligned}&E_0^{-}(u_{\infty }^{-},v_{\infty }^{-},q_{\infty }^{-},w_{\infty }^{-}) = \\ &\left( u_{\infty }^{-}, 0, 1-\Big (\dfrac{u_{\infty }^{-}}{u_0}\Big )^{\gamma /{\mathscr {R}}_0}, \Big (\dfrac{u_{\infty }^{-}}{u_0}\Big )^{\gamma /{\mathscr {R}}_0}-u_{\infty }^{-} \right) , \end{aligned}$$

with a positive susceptible subpopulation size $u_{\infty }^{-}\in (0, u_0)$, which is determined by the unique positive root of equation

12 $$\begin{aligned} u_{\infty }^{-}=F(u_{\infty }^{-}). \end{aligned}$$

Equation (12) is derived by taking $\tau \rightarrow \infty$ for the equality (7) with $v\rightarrow 0$. The disease-eliminated equilibrium $E_0^{-}$ is uniquely determined for each given initial condition with $u_0> 0$. In other words, the disease-eliminated equilibrium $E_0^{-}$ depends on the initial condition given by the initial infective subpopulation size $v_0$ (alternatively $u_0$).

In the next section, we will show the necessary and sufficient condition that the system (5) always remains at the isolation effective phase, and alternatively the condition that the isolation reaches the capacity at finite time along the path of the epidemic process. As an important preliminary found by the arguments in Appendix C for Theorem 5.1, we obtain the following lemma too:

### Lemma 5.3

The system (5) can always remain at the isolation effective phase only if $\varepsilon {\mathscr {R}}_0 < 1$. Otherwise, if $\varepsilon {\mathscr {R}}_0\ge 1$, then the isolation reaches the capacity, and the system enters the isolation incapable phase at finite time.

This result shows a sufficient condition that the isolation reaches the capacity at finite time. Even when $\varepsilon {\mathscr {R}}_0< 1$, there could be such a case as shown in the next section.

## Condition for the isolation incapable phase

Taking account of the results shown in the previous section, we can prove the following theorem to show the necessary and sufficient condition that the isolation reaches the capacity at finite time along the path of the epidemic process (Appendix D):

### Theorem 6.1

Isolation reaches the capacity and becomes incapable at finite time along the path of the epidemic process if and only if one of the following conditions is satisfied:

(i) $$\varepsilon {\mathscr {R}}_0\ge 1$$;
(ii) $$\varepsilon {\mathscr {R}}_0< 1$$ and
  13 $$\begin{aligned} u_0 (1-q_{\textrm{max}} )^{{\mathscr {R}}_0/\gamma } < F\big ( u_0 (1-q_{\textrm{max}} )^{{\mathscr {R}}_0/\gamma } \big ). \end{aligned}$$

Otherwise, if both conditions (*i*) and (*ii*) are not satisfied, the isolation never reaches the capacity in the epidemic dynamics.

In other words, the system always remains at the isolation effective phase when and only when both conditions (*i*) and (*ii*) are not satisfied. The inequality (13) for $\varepsilon = 0$ matches the condition obtained in Ahmad and Seno (2023) on the SIR+Q model without reinfection.

This result of Theorem 6.1 can be translated in the following way with the critical value $q_c$ for the isolation capacity $q_{\textrm{max}}$ (Appendix D):

### Corollary 6.1.1

Isolation reaches the capacity and becomes incapable at finite time if and only if $q_{\textrm{max}}<q_{c}$, where $q_{c}$ is defined as the smallest positive root of equation

14 $$\begin{aligned} u_0 (1-q_{c} )^{{\mathscr {R}}_0/\gamma }=F\big (u_0 (1-q_{c} )^{{\mathscr {R}}_0/\gamma }\big ). \end{aligned}$$

If and only if $q_{\textrm{max}}\ge q_{c}$, the system always remains at the isolation effective phase, where the isolation never reaches the capacity.

**Fig. 3:**
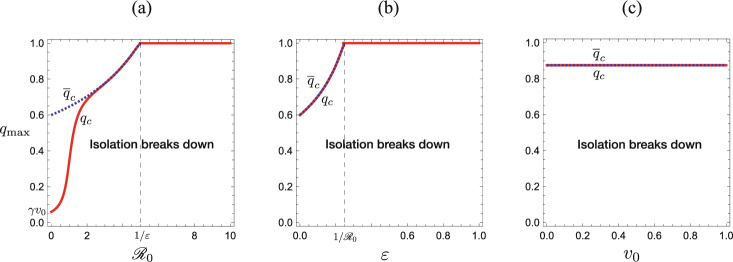
**a** ${\mathscr {R}}_0$-dependence; **b** $\varepsilon$-dependence; **c** $v_0$-dependence of the critical value $q_c$ of the isolation capacity $q_{\textrm{max}}$. Numerically drawn by Theorem 6.1, Corollaries 6.1.1 and 6.1.2 with **a** $\varepsilon = 0.2$, $u_0=0.9$; **b** ${\mathscr {R}}_0 = 4$, $u_0=0.9$, **c** $\varepsilon = 0.2$, ${\mathscr {R}}_0 = 4$, and commonly $\gamma =0.6$. The boundary $q_c$ is given by (14), and the dotted curve of ${\overline{q}}_c$ is by (15). The difference between $q_c$ and ${\overline{q}}_c$ appears rather slight in (**b**) and (**c**). In (**c**), ${\overline{q}}_c = 0.875$ independently of $v_0$ while $q_c$ depends on $v_0 = 1-u_0$

As shown in Appendix D and Fig. 3, the critical value $q_c$ defined in Corollary 6.1.1 becomes less than 1 for $\varepsilon {\mathscr {R}}_0 <1$, while it becomes 1 for $\varepsilon {\mathscr {R}}_0 \ge 1$. Since $q_{\textrm{max}}< 1$ from our assumption, it is impossible to satisfy that $q_{\textrm{max}}\ge q_{c}$ when $\varepsilon {\mathscr {R}}_0 \ge 1$. That is, the system necessarily enters the isolation incapable phase at finite time, in accordance with the result shown in Theorem 6.1.

From equation (14), we can easily find that the critical value $q_c$ for the isolation capacity $q_{\textrm{max}}$ is monotonically increasing in terms of the basic reproduction number ${\mathscr {R}}_0$, the index for the reinfection $\varepsilon$, and the initial infective subpopulation size $v_0$. The stronger infectivity, the higher likelihood of reinfection, or the larger number of the initial infected individuals leads to the demand of a larger isolation capacity to avoid its breakdown in the epidemic dynamics, as numerically illustrated in Fig. 3.

Moreover, we note that $q_c\rightarrow \gamma v_0 =\gamma (1-u_0)$ as ${\mathscr {R}}_0\rightarrow 0$ with (14) (see Fig. 3a). We can easily find that the condition (13) becomes $q_{\textrm{max}}<\gamma (1-u_0)$ as ${\mathscr {R}}_0\rightarrow 0$. This is a reasonable mathematical feature about our model (5). When no disease transmission occurs with ${\mathscr {R}}_0 = 0$, every initial infective belonging to $v_0$ alternatively recovers or is isolated, and the system eventually approaches the equilibrium $(u, v, q, w) = (u_0, 0, \gamma v_0, (1-\gamma )v_0)$, if the isolation capacity is not below $\gamma v_0$, which can be easily found by considering the system (5) with ${\mathscr {R}}_0 = 0$. Otherwise, if $q_{\textrm{max}}<\gamma v_0$, the isolation reaches the capacity on the way of the infective elimination, and it becomes incapable.

As clearly indicated by Theorem 6.1, the isolation reaches the capacity at finite time if $q_{\textrm{max}}<q_{c}$ even when $\varepsilon {\mathscr {R}}_0< 1$. It has been already shown in Lemma 5.3 that the system can always remain at the isolation effective phase only when $\varepsilon {\mathscr {R}}_0< 1$, and now we can find the following subsidiary result too (Appendix D):

### Corollary 6.1.2

When $$\varepsilon {\mathscr {R}}_0< 1$$, if

15 $$\begin{aligned} q_{\textrm{max}} \ge {\overline{q}}_c := \left\{ \begin{array}{ll} 1- \Big (\dfrac{1-\varepsilon {\mathscr {R}}_0}{1-\gamma }\Big )^{\gamma / (\varepsilon {\mathscr {R}}_0-\gamma )} & \text{ for }\ \varepsilon {\mathscr {R}}_0\ne \gamma ; \\ 1-\mathrm e^{-\gamma /(1-\gamma )} & \text{ for }\ \varepsilon {\mathscr {R}}_0= \gamma , \end{array} \right. \end{aligned}$$

the system always remains at the isolation effective phase and the isolation never reaches the capacity.

This corollary gives a sufficient condition that the system always remains at the isolation effective phase when $\varepsilon {\mathscr {R}}_0< 1$, that is, the right side of (15) gives a sufficient isolation capacity for it, independently of the initial condition given by the value $u_0$. The sufficient isolation capacity ${\overline{q}}_c$ is the supremum of $q_c$ in terms of $u_0$: It holds that ${\overline{q}}_c> q_c$, so that we have $q_{\textrm{max}}>q_c$ if $q_{\textrm{max}}\ge {\overline{q}}_c$ (see Fig. 3). Only if the condition (15) is unsatisfied, the system enters the isolation incapable phase at finite time along the path of the epidemic process.

As the other important subsidiary result obtained in the proof for Corollary 6.1.1 in Appendix D, we can find

### Lemma 6.1

$$u_{\infty }^{-}=u_0(1-q_c)^{{\mathscr {R}}_0/\gamma }$$.

This result will be useful in the subsequent analysis. Note that the equilibrium value $u_\infty ^-$ is independent of the isolation capacity $q_{\textrm{max}}$ because it is for the equilibrium at the isolation effective phase when the isolation never reaches the capacity.

## Revival of outbreak

As already seen in Fig. 2b, c, there could be a case where the infective subpopulation size turns from decreasing to increasing at the moment that the isolation reaches the capacity and the system enters the isolation incapable phase. Such a case appears as a revival of outbreak of the disease spread in the community. We can get the following condition that such a revival of outbreak occurs (Appendix E):

### Theorem 7.1

When the isolation reaches the capacity at finite time, a revival of outbreak occurs if

$$\begin{aligned}&\dfrac{\varepsilon {\mathscr {R}}_0-1}{{\mathscr {R}}_0}(1-q_{\textrm{max}})<\varepsilon F(u^{\star }) -u^{\star } \\&\quad <\dfrac{\varepsilon {\mathscr {R}}_0-(1-\gamma )}{{\mathscr {R}}_0}(1-q_{\textrm{max}}), \end{aligned}$$

where $u^\star$ is defined by (10).

**Fig. 4:**
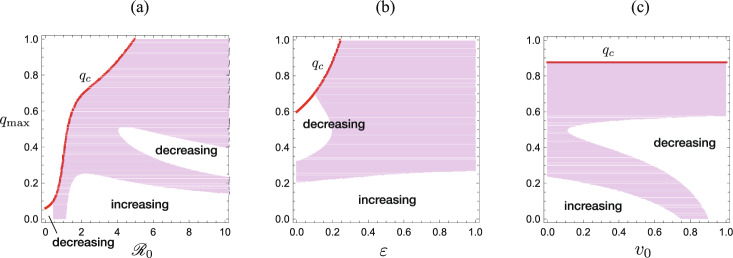
Parameter region for the revival of outbreak, numerically drawn by Theorem 7.1 with the parameter values used in Fig. 3: **a** ${\mathscr {R}}_0$-dependence; **b** $\varepsilon$-dependence; **c** $v_0$-dependence. The revival of outbreak occurs for the filled region. For the other region, the infective subpopulation size keeps decreasing or increasing even at the moment that the isolation reaches the capacity and the system enters the isolation incapable phase

Figure 4 shows numerically obtained parameter regions for the revival of outbreak. It is implied that the parameter dependence is not simple. Roughly the larger isolation capacity or the larger infectivity is more likely to cause the revival of outbreak, while the sufficiently small isolation capacity is less likely. We may expect that the breakdown of the isolation operation along the path of the epidemic process could lead to the revival of outbreak.

## Equilibria for the isolation incapable phase

We obtain the following lemma and theorem about the feasible equilibria at the isolation incapable phase (Appendix F):

### Lemma 8.1

At the isolation incapable phase, $u\rightarrow u_\infty ^+\in (0, u^\star )$ as $\tau \rightarrow \infty$, if and only if $\varepsilon {\mathscr {R}}_0 < 1-\gamma$. The equilibrium value $u_\infty ^+$ is determined by the unique positive root in $(0, u^\star )$ of equation

16 $$\begin{aligned} u_{\infty }^{+}=G(u_{\infty }^{+}), \end{aligned}$$

where *G* is defined by (8) and (9). If $\varepsilon {\mathscr {R}}_0 \ge 1-\gamma$, then $u\rightarrow 0$ as $\tau \rightarrow \infty$.

### Theorem 8.1

At the isolation incapable phase, if and only if $\varepsilon {\mathscr {R}}_0< 1-\gamma$, the system (5) approaches a disease-eliminated equilibrium $E_0^{+}$:

17 $$\begin{aligned} E_0^{+}(u_{\infty }^{+},v_{\infty }^{+},q_{\infty }^{+},w_{\infty }^{+}) = \big (u_{\infty }^{+},0,q_{\textrm{max}}, 1-u_{\infty }^{+}-q_{\textrm{max}} \big ), \end{aligned}$$

where $u_\infty ^+$ is determined by the unique positive root in $(\, 0, u^\star )$ of equation (16). If $\varepsilon {\mathscr {R}}_0 = 1-\gamma$ at the isolation incapable phase, it approaches the disease-eliminated equilibrium $E_0^{+}$ given as $(0, 0, q_{\textrm{max}}, 1-q_{\textrm{max}})$. Otherwise, if $\varepsilon {\mathscr {R}}_0> 1-\gamma$ at the isolation incapable phase, it approaches the endemic equilibrium $E_*^+$:

18 $$\begin{aligned}&E_*^+(u_{\infty }^{+},v_{\infty }^{+},q_{\infty }^{+},w_{\infty }^{+}) =\nonumber\\ &\left( \ 0,\, \Big ( 1-\frac{1-\gamma }{\varepsilon {\mathscr {R}}_0} \Big )(1-q_{\textrm{max}}),\, q_{\textrm{max}}, \frac{1-\gamma }{\varepsilon {\mathscr {R}}_0} (1-q_{\textrm{max}})\, \right) . \end{aligned}$$

As a result, the system approaches an endemic equilibrium if and only if $\varepsilon {\mathscr {R}}_0> 1-\gamma$ at the isolation incapable phase. Otherwise, it approaches a disease-eliminated equilibrium, independently of whether it enters the isolation incapable phase or not.

Figure 2b, c numerically exemplifies the cases in which the system approaches an disease-eliminated equilibrium $E_0^{+}$ and the endemic equilibrium $E_*^+$, respectively, after it enters the isolation incapable phase. The endemic state arises in the community necessarily after the isolation reaches the capacity. The endemic state is sustained by the reinfection for the recovered individuals, since there is no susceptible who has not experienced the disease in the community (i.e., $u_\infty ^+ = 0$). From Theorems 5.1 and 8.1, even after the isolation reaches the capacity, the elimination of the disease may occur if the reinfectivity is weak enough to satisfy that $\varepsilon {\mathscr {R}}_0 \le 1-\gamma$.

## Endemic size

The endemic size is defined here as the equilibrium value of the infective subpopulation size $v_\infty$, which is hence zero if the system approaches a disease-eliminated equilibrium. From Theorem 8.1, it can become positive only at the isolation incapable phase, and given as $v_\infty ^+ = \{ 1-(1-\gamma )/(\varepsilon {\mathscr {R}}_0)\}(1-q_{\textrm{max}})$ from $E_*^+$ given by (18).

**Fig. 5:**
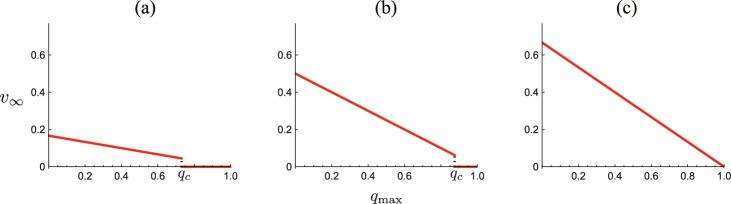
$q_{\textrm{max}}$-dependence of the endemic size $v_\infty$. Numerically drawn with **a** $\varepsilon = 0.12$ ($\varepsilon {\mathscr {R}}_0 = 0.48$; $q_c = 0.7305$); **b** $\varepsilon = 0.2$ ($\varepsilon {\mathscr {R}}_0 = 0.8$; $q_c = 0.8750$); **c** $\varepsilon = 0.3$ ($\varepsilon {\mathscr {R}}_0 = 1.2$; $q_c = 1$), and commonly $u_0=0.9$; $\gamma = 0.6$; ${\mathscr {R}}_0 = 4.0$. Note that $v_\infty = 0$ independently of $q_{\textrm{max}}$ if $\varepsilon {\mathscr {R}}_0\le 1-\gamma$, as indicated in Theorem 8.1

From Theorem 6.1, Corollary 6.1.1, and Theorem 8.1, we have noted that, when $\varepsilon {\mathscr {R}}_0 \ge 1$, the system necessarily approaches an endemic equilibrium at the isolation incapable phase. Then, the endemic size $v_\infty ^+$ is monotonically decreasing in terms of the isolation capacity $q_{\textrm{max}}$ as shown by (18). See the numerical example in Fig. 5c.

In contrast, especially when the disease spreads with $\varepsilon {\mathscr {R}}_0 \in (1-\gamma , 1\, )$, the disease becomes eliminated if $q_{\textrm{max}}\ge q_c\in (0, 1)$, while it becomes endemic if $q_{\textrm{max}}< q_c$, as seen in the numerical examples of Fig. 5a, b. Then, the endemic size shows a discontinuity at $q_{\textrm{max}}= q_c$, where it is continuous and positive in terms of $q_{\textrm{max}}< q_c$, and zero for $q_{\textrm{max}}> q_c$. Thus, in such a case, the isolation capacity is a crucial factor for the endemicity of the spreading disease.

## Final epidemic size

The final epidemic size $z_{\infty }$ is defined here as the proportion of individuals in the community who have experienced the infection until the final stage of the epidemic dynamics. Hence, it is given by $z_{\infty }:=1-u_{\infty }$ for the system (5). From this definition of the final epidemic size, when the system (5) approaches an endemic equilibrium, we have $z_\infty = 1$, because every individual in the community has experienced the infection at the end of the epidemic dynamics with $u_\infty = 0$.

First, as shown by Theorems 6.1, 8.1, and Corollary 6.1.1, when the isolation never reaches the capacity in the epidemic dynamics with $\varepsilon {\mathscr {R}}_0<1$ and $q_{\textrm{max}}\ge q_{c}$, the system (5) approaches a disease-eliminated equilibrium, and then the final epidemic size $z_\infty$ is given by $z_\infty ^-:=1-{u^{-}_\infty }$ with $u^{-}_\infty$ given by Lemma 6.1. That is, we have $z_{\infty }^{-} = 1-u_0(1-q_c)^{{\mathscr {R}}_0/\gamma } \in (0, 1)$.

Next, from those results obtained in the previous sections, when the isolation reaches the capacity at finite time with $q_{\textrm{max}}<q_{c}$, we have the following results on the final epidemic size $z_\infty$:

- If $\varepsilon {\mathscr {R}}_0< 1-\gamma$, the system (5) approaches a disease-eliminated equilibrium as shown by Theorem 8.1. Then, the final epidemic size $z_\infty$ is given by $z_\infty ^+:=1-u_\infty ^+$ with the unique positive root $u^{+}_\infty$ of equation (16).
- If $\varepsilon {\mathscr {R}}_0= 1-\gamma$, the system (5) approaches a disease-eliminated equilibrium accompanied with $u\rightarrow 0$ as $\tau \rightarrow \infty$, as shown by Theorem 8.1. Then, the final epidemic size $z_\infty$ is given by $z_\infty ^+=1$.
- If $\varepsilon {\mathscr {R}}_0> 1-\gamma$, the system (5) approaches the endemic equilibrium (18) as shown by Theorem 8.1. Then, the final epidemic size becomes $z_\infty =1$ accompanied with $u\rightarrow 0$ as $\tau \rightarrow \infty$.

**Fig. 6:**
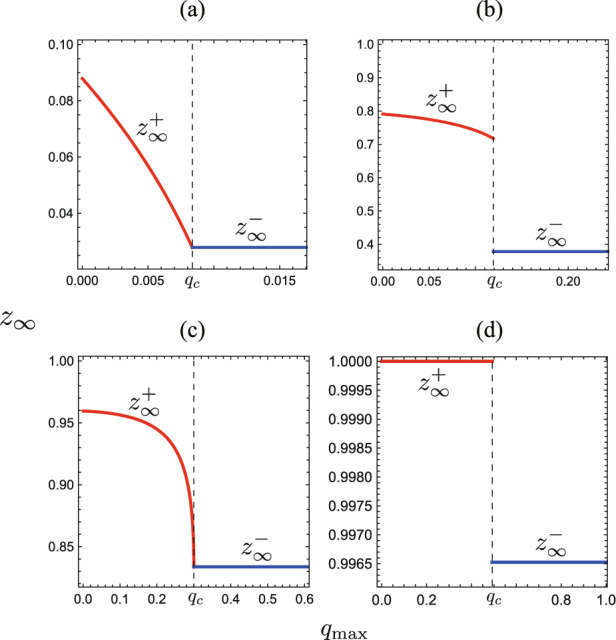
$q_{\textrm{max}}$-dependence of the final epidemic size $z_\infty$. Numerically drawn for **a** ${\mathscr {R}}_0 = 0.65$ ($\varepsilon {\mathscr {R}}_0 = 0.195$; $q_c = 0.0084$); **b** ${\mathscr {R}}_0 = 1.1$ ($\varepsilon {\mathscr {R}}_0 = 0.33$; $q_c = 0.1192$); **c** ${\mathscr {R}}_0 = 1.5$ ($\varepsilon {\mathscr {R}}_0 = 0.45$; $q_c = 0.3000$); **d** ${\mathscr {R}}_0 = 2.5$ ($\varepsilon {\mathscr {R}}_0 = 0.75$; $q_c = 0.4925$), and commonly $u_0=0.99$; $\varepsilon = 0.3$; $\gamma = 0.3$. In (**d**), $z_\infty ^+ = 1$ because the system approaches an endemic equilibrium with $\varepsilon {\mathscr {R}}_0> 1-\gamma$ as indicated in Theorem 8.1

Especially as for the final epidemic size at the isolation incapable phase $z_\infty = z_\infty ^+$ with $\varepsilon {\mathscr {R}}_0< 1-\gamma$ and $q_{\textrm{max}}<q_{c}$, we can find the following feature (Appendix G):

### Lemma 10.1

The final epidemic size $z_\infty = z_\infty ^+$ is monotonically decreasing in terms of $q_{\textrm{max}}\in (0, q_c)$ at the isolation incapable phase with $\varepsilon {\mathscr {R}}_0< 1-\gamma$.

Figure 6 numerically shows the $q_{\textrm{max}}$-dependence of the final epidemic size $z_\infty$. It is seen that the larger isolation capacity makes the final epidemic size smaller. Figure 6b shows a case where the final epidemic size $z_\infty$ becomes drastically large if the isolation reaches the capacity at finite time. The same tendency is seen also in Fig. 6d, whereas the difference between $z_\infty ^-$ (around 0.9965) and $z_\infty ^+ = 1$ is rather small. In contrast, the final epidemic size $z_\infty$ can be continuous in terms of the isolation capacity $q_{\textrm{max}}$ as shown in Fig. 6a, c.

We can obtain the following analytical result on such the discontinuity in the $q_{\textrm{max}}$-dependence of the final epidemic size $z_\infty$ (Appendix H):

### Theorem 10.1

When $\varepsilon {\mathscr {R}}_0 < 1$, the final epidemic size $z_\infty$ has a discontinuity at $q_{\textrm{max}}=q_{c}$ such that

$$\begin{aligned} z^{\dagger }_{\infty } := \lim _{q_{\textrm{max}} \rightarrow q_{c}-0} z_\infty ^+> z_\infty ^- \end{aligned}$$

if and only if one of the following conditions is satisfied:

(i) $$1-\gamma \le \varepsilon {\mathscr {R}}_0<1$$;
(ii) $$\varepsilon (1-\gamma )< \varepsilon {\mathscr {R}}_0 < 1-\gamma$$ and
  19 $$\begin{aligned} u_0(1-q_c)^{{\mathscr {R}}_0/\gamma -1}> \dfrac{\varepsilon }{1-\varepsilon }\Big (\dfrac{1-\gamma }{\varepsilon {\mathscr {R}}_0}-1\Big ). \end{aligned}$$

If the condition (19) is unsatisfied for $\varepsilon {\mathscr {R}}_0 \in (\varepsilon (1-\gamma ), 1-\gamma )$, then it holds that $z^{\dagger }_{\infty }=z_\infty ^-$.

The condition (ii) for $\varepsilon = 0$ becomes coincident with the condition obtained in Ahmad and Seno (2023) for such a discontinuity about the SIR+Q model without reinfection. The numerical example Fig. 6d shows the case (i) in Theorem 10.1, and Fig. 6b does the case (ii). Figure 6c shows the case where the condition (19) is unsatisfied with $\varepsilon {\mathscr {R}}_0 \in (\varepsilon (1-\gamma ), 1-\gamma )$. In contrast, Fig. 6a corresponds to the case of ${\mathscr {R}}_0<1-\gamma$.

**Fig. 7:**
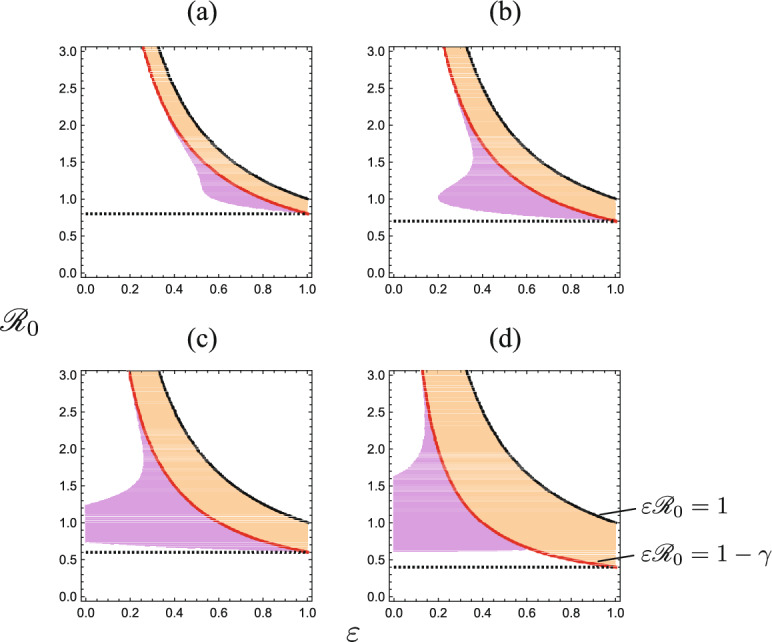
($\varepsilon , {\mathscr {R}}_0$)-dependence of the discontinuity of the final epidemic size $z_\infty$ at $q_{\textrm{max}}=q_{c}$. For the filled region, we have $z_\infty ^\dagger> z_\infty ^-$ as shown in Theorem 10.1. Numerically drawn with **a** $\gamma = 0.2$; **b** $\gamma = 0.3$; **c** $\gamma = 0.4$; **d** $\gamma = 0.6$, and commonly $u_0 = 0.9$. The upper solid boundary curve is of $\varepsilon {\mathscr {R}}_0 = 1$, and the lower is of $\varepsilon {\mathscr {R}}_0 = 1-\gamma$. The horizontal dotted line is of ${\mathscr {R}}_0 = 1-\gamma$. The detail is in the main text

In Fig. 7, we numerically show the $(\varepsilon , {\mathscr {R}}_0)$-dependence of the discontinuity of the final epidemic size $z_\infty$ at $q_{\textrm{max}}=q_{c}$. For the region corresponding to the case (i) in Theorem 10.1, that is, for the region between two solid boundary curves $\varepsilon {\mathscr {R}}_0 = 1$ and $\varepsilon {\mathscr {R}}_0 = 1-\gamma$, we have an endemic equilibrium (18) with $z_\infty ^+ = 1$ for $q_{\textrm{max}}<q_{c}$, when we can observe the discontinuity at $q_{\textrm{max}}=q_{c}$ as Fig. 6d. For the filled region below the solid boundary curve $\varepsilon {\mathscr {R}}_0 = 1-\gamma$, corresponding to the case (ii) in Theorem 10.1, we have a disease-eliminated equilibrium (17) with $z_\infty ^+ < 1$ for $q_{\textrm{max}}> q_{c}$, when we can observe the discontinuity at $q_{\textrm{max}}=q_{c}$ as Fig. 6b. For the blank region below the solid boundary curve $\varepsilon {\mathscr {R}}_0 = 1-\gamma$ in Fig. 7, we have $z_\infty ^+\rightarrow z_\infty ^-$ as $q_{\textrm{max}}\rightarrow q_{c}-0$, when the final epidemic size $z_\infty$ is continuous even at $q_{\textrm{max}}=q_{c}$ as Fig. 6a. For the blank region beyond the solid boundary curve $\varepsilon {\mathscr {R}}_0 = 1$ in Fig. 7, we have an endemic equilibrium (18) at the isolation incapable phase for any $q_{\textrm{max}}\in [0, 1)$, and there is no case of $q_{\textrm{max}} \ge q_c = 1$. As indicated by Fig. 7, although not simple is the dependence of the discontinuity of the final epidemic size $z_\infty$ on the nature of spreading disease, represented by the parameters ${\mathscr {R}}_0$ and $\varepsilon$, it is implied that the higher risk of reinfection (i.e., with the larger $\varepsilon$) is more likely to cause such the discontinuity. Moreover, the faster isolation (i.e., with the larger $\gamma$) is more like to do so too. The faster isolation means the more effective quarantine, which could be regarded as a better feature in the isolation operation for the public health measure. Therefore, sufficiently effective quarantine and fast isolation would be highly important to suppress the endemic size or the final epidemic size, because the sufficient capacity of isolation may drastically reduce such sizes as the consequence of the epidemic dynamics.

## Dependence on the quarantine efficiency

**Fig. 8:**
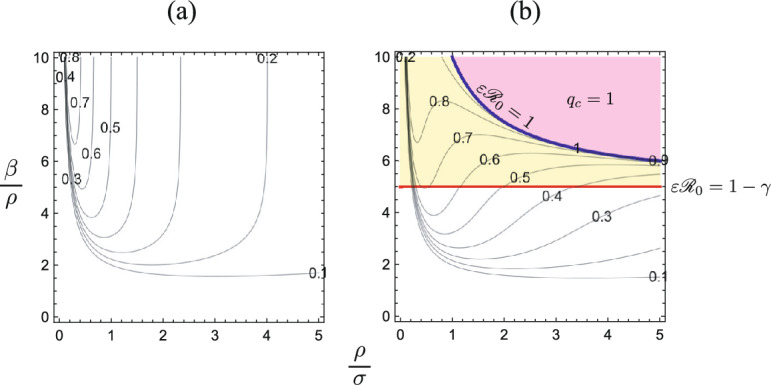
Numerically drawn contour plots of $q_c$ in terms of ($\rho /\sigma , \beta /\rho$), making use of Theorem 6.1, Corollary 6.1.1, and Theorem 8.1: **a** $\varepsilon = 0$; **b** $\varepsilon = 0.2$. Commonly, $u_0=0.99$. In (**b**), the endemic equilibrium may appear only for $\varepsilon {\mathscr {R}}_0> 1-\gamma$, that is, for $\beta /\rho> 1/\varepsilon$. For $\varepsilon {\mathscr {R}}_0 \ge 1$, that is, for $\varepsilon \beta /\rho \ge 1+\sigma /\rho$, the system (5) necessarily enters the isolation incapable phase for any isolation capacity $q_{\textrm{max}}$, corresponding to $q_c = 1$

Figure 8 shows the numerical calculation of the $(\rho /\sigma , \beta /\rho )$-dependence of $q_c$ for our model (5), where we used $q_c$ determined by (14) in Corollary 6.1.1, which can be expressed by only four parameters $u_0$, $\varepsilon$, $\beta /\rho$, and $\rho /\sigma$ with the original parameters in our model (4). The parameter $\beta /\rho$ corresponds in fact to the basic reproduction number of the epidemic dynamics by (4) without isolation. Hence, in contrast to ${\mathscr {R}}_0$ with isolation, we may call $\beta /\rho$ the *primitive* basic reproduction number at the stage of the disease invasion in the community when the quarantine measure has not yet been applied.

Numerical results in Fig. 8 clearly demonstrate that the severe epidemic with the larger primitive basic reproduction number requires the larger isolation capacity to avoid its breakdown, which matches our intuitive expectation as seen in the ${\mathscr {R}}_0$-dependence of $q_c$ in Sect. 6 (refer to Fig. 3). On the whole, a sufficiently high efficiency of the quarantine could make the isolation capacity smaller to avoid its breakdown, and a sufficiently low efficiency could induce the breakdown, as mentioned also at the end of the previous section. On the other hand, the critical isolation capacity $q_c$ appears to have a nontrivial relation to the efficiency of quarantine operation, represented by the parameter $\sigma$. There are some cases where $q_c$ becomes relatively large in an intermediate range of $\sigma$, while $q_c$ gets smaller for sufficiently small or large $\sigma$. Such a nontrivial dependence of the $q_c$ on the quarantine efficiency was found and discussed also in Ahmad and Seno (2023) on the SIR+Q model without reinfection (refer to Fig. 8a). Our numerical calculations in Fig. 8 imply that such a feature appears remarkably for $\varepsilon = 0$, that is, for the model without reinfection, and it becomes more complicated for the model with reinfection.

**Fig. 9:**
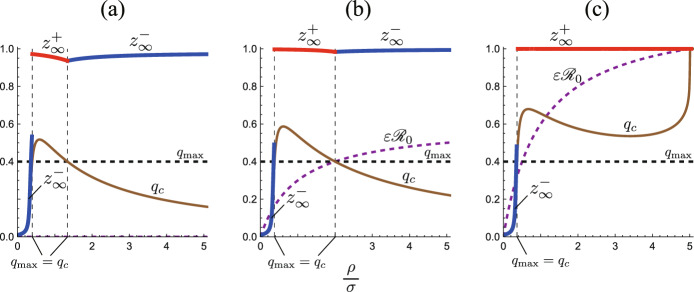
$\rho /\sigma$-dependence of the critical isolation capacity $q_c$ and the final epidemic size $z_\infty$. Numerically drawn for **a** $\varepsilon = 0.0$; **b** $\varepsilon = 0.15$; **c** $\varepsilon = 0.30$, and commonly $u_0=0.99$; $q_{\textrm{max}}=0.4$; $\beta /\rho = 4.0$

Actually as for the dependence of the final epidemic size $z_\infty$ on the quarantine efficiency, it can result in an unexpected feature as shown in Fig. 9. As Ahmad and Seno (2023) investigated for the model without reinfection, the final epidemic size $z_\infty$ could have a non-monotone relation to $\sigma$, and then $z_\infty$ could take a local maximum for an intermediate value of $\sigma$. Sufficiently high quarantine efficiency (i.e., sufficiently large $\sigma$) can make the critical isolation capacity $q_c$ rather small, and thus it can significantly reduce the final epidemic size $z_\infty$. As indicated by Fig. 9, it is necessary for the quarantine to have a sufficiently high efficiency in order to avoid the breakdown of isolation and to successfully suppress the final epidemic size. However, as shown by the numerical calculations in Figs. 8 and 9, the reinfection could make complicated the relation of the quarantine efficiency to the critical isolation capacity, and such a complicatedness implies a difficulty to prepare an appropriate measure of the quarantine and isolation for the public health in a community.

From the definition of parameters $\rho$ and $\sigma$ for the epidemic dynamics by (4), the expected duration of the infectivity (i.e., the transmissibility of the disease by an infective) is given by $1/\rho$, and the expected duration of the detection of an infective until it gets isolated is given by $1/\sigma$ at the isolation effective phase. In a sense, it would be reasonable to assume that $1/\sigma < 1/\rho$, that is, $\rho /\sigma < 1$, because the detection of an infective is possible only when the individual has the infectivity. However, the quarantine efficiency must depend on the availability of medical services and the voluntary access of infectives to such a service. Therefore, with the dependence on such factors, poor quarantine efficiency could make $\rho /\sigma \ge 1$.

## Concluding remarks

Results on our mathematical model clearly indicate that the increase in the isolation capacity makes the endemic size and the final epidemic size smaller as implied by the mathematical results on the SIR+Q model in Ahmad and Seno (2023), while mathematical arguments required to show important features are rather different from those in their work because of the reinfection introduced in our model. More significantly, it is implied that the breakdown of isolation due to its limited capacity could induce a considerable change of the epidemic severity accompanied with the revival of outbreak, the emergence of endemicity, or a staggeringly wide spread of the disease, for example. In other words, the isolation capacity could be a crucial factor for the public health policy not only to reduce the epidemic size but also to suppress the endemicity.

**Fig. 10:**
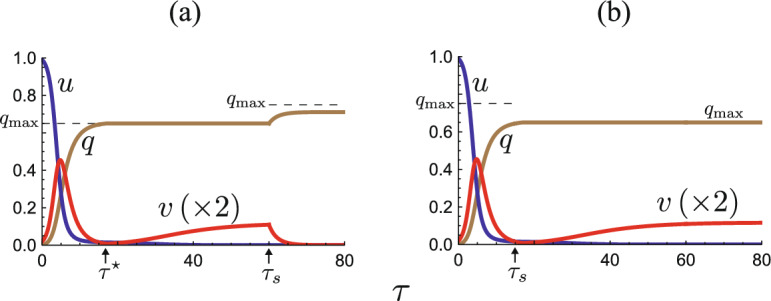
Numerical examples for the temporal variation of the model (5) with a change of the isolation capacity at $\tau =\tau _s$: **a** $q_{\textrm{max}}=0.65$ to 0.75, $\tau _s =60$; **b** $q_{\textrm{max}}=0.75$ to 0.65, $\tau _s =15$. Commonly, $\gamma = 0.5$; ${\mathscr {R}}_0 = 2.0$; $\varepsilon = 0.3$ ($\varepsilon {\mathscr {R}}_0 = 0.6$); $u_0=0.99$; $q_c = 0.6554$. In (**a**), the isolation reaches the capacity and becomes incapable at a moment $\tau = \tau ^\star$, and then the disease tends to become endemic until $\tau = \tau _s$, whereas it turns to be eliminated after the raise of isolation capacity at $\tau =\tau _s$. In (**b**), the disease tends to be eliminated until $\tau = \tau _s$, whereas it revives after the reduction of isolation capacity after it

The higher risk of reinfection leads to the larger critical capacity of isolation: The larger isolation capacity is necessary to avoid the severe consequence of the epidemic dynamics with a reinfectious disease. In general, the reinfectivity of spreading disease must induce the higher importance of the isolation capacity for the effective public health measure, because the recovered individual may get infected again and further become a spreader of the disease. Actually, since the existence of reinfectivity could induce the endemicity of the disease, the isolation capacity must be rather important to control the disease spread. Figure 10 gives numerical examples with our model (5) to indicate the importance. An increase in the isolation capacity may result in the effective suppression of the endemicity and drive the disease to its elimination as in Fig. 10a. In contrast, a careless reduction of the isolation capacity, as in Fig. 10b, for example, because of the low prevalence monitored in the epidemic dynamics, may induce the revival of the disease spread by the released endemicity with the reinfectivity.

In our results as illustrated by Fig. 7, when the isolation capacity is insufficient, the higher risk of reinfection is more likely to not only induce an endemic state but also lead to a discontinuously larger epidemic size even though the disease finally gets eliminated. Further, as was shown in our mathematical results, the highly effective quarantine with a sufficient capacity of isolation could result in a successful suppression of the endemic size or the final epidemic size to an unexpectedly distinct extent. This implies the importance of the isolation capacity as a measure for the public health, while such a sufficient capacity of the isolation or an effective quarantine must be ready before the outbreak of a disease spread because it would generally become hard to prepare after it (Baker et al. 2023).

The smaller critical value of the isolation capacity $q_c$ is better for the management of the epidemic dynamics. That is, the smaller critical value for the isolation capacity makes an isolation policy with a feasible capacity more likely to be invulnerable to avoid its breakdown. The larger critical value for the isolation capacity indicates a harder situation for the public health policy since a large capacity of isolation is necessary to avoid its breakdown and to suppress the endemicity or make the final epidemic size at a low level. As the factors to determine the effectiveness of a public health policy against a spreading disease, the isolation capacity and the quarantine efficiency could be independently improved. Our results clearly indicate their relevance, and it is implied that the improvement about one of them could make that about the other more feasible, as discussed in Shahverdi et al. (2023). Inversely, when one of them could not be sufficiently improved, the improvement of the other becomes less effective.

Naturally the quarantine/isolation could not be necessarily the principal factor for the public health policy against the spread of an infectious disease, while it must be important and could have a significant contribution to the suppression of the epidemic size, accompanied with the other measures against the epidemic. Our theoretical results would highlight the importance of satisfactory infrastructure for the public health as indicated by Unruh et al. (2022) on the social response to the COVID-19 pandemic. Since the satisfactory infrastructure for the public health needs a sufficient social investment, these arguments on our model would imply a difficulty of the management of even quarantine/isolation policy against an infectious disease spreading in a community too.

In our modeling, we assumed no discharge from the isolation. However, in most real cases, it may occur even to make effective the use of available medical resources (space, medical equipments, medical workers, etc.), and very probably also in the epidemic dynamics with a reinfectious disease. However, such a discharge of recovered individuals from the isolation could serve a supply of potential hosts for the disease spread because of its reinfectivity. In this sense, the discharge rate must be one of the important factors to determine the effectivity of quarantine/isolation measure for the public health. It is worth considering the model with such a discharge of recovered individuals from the isolation which has a limited capacity. We would like to study further such a model elsewhere in future, for example, concerning the efficiency of lockdown policy or self-responsible isolation according to the suppression of epidemic size.

## Data Availability

Not applicable.

## Appendix A: Proof for Lemma 3.1

Note that the isolated population size *q* never becomes greater than $q_{\textrm{max}}$ in the epidemic dynamics governed by (5) with the initial value $q(0) = 0$. From (5), we have $\hbox {d}q/\hbox {d}\tau = 0$ for $q= q_{\textrm{max}}$. Thus, also in accordance with the modeling assumption, the isolated population size *q* never becomes greater than $q_{\textrm{max}}< 1$ for any $\tau \ge 0$.

Then from (5), we formally have

$$\begin{aligned} v(\tau ) = v(0)\exp \left[ \int _0^\tau {\mathscr {R}}_0\dfrac{u(\nu )}{1-q (\nu )}+\varepsilon {\mathscr {R}}_0\dfrac{w(\nu )}{1-q (\nu ) } \, d\nu -\tau \, \right]> 0 \end{aligned}$$

with $v(0)> 0$ for any $\tau$ such that $q(\nu )<q_{\textrm{max}}$ for any $\nu < \tau$. If the system gets in the isolation incapable phase after $\tau = \tau ^\star> 0$, we have $v(\tau ^\star )> 0$ from the above argument, and

$$\begin{aligned} v(\tau )&= v(\tau ^\star )\exp \left[ \int _{\tau ^\star }^\tau {\mathscr {R}}_0\dfrac{u(\nu )}{1-q (\nu )}\right. \\&\quad \left. +\varepsilon {\mathscr {R}}_0\dfrac{w(\nu )}{1-q (\nu ) } \, d\nu -(1-\gamma )(\tau -\tau ^\star )\, \right]> 0 \end{aligned}$$

for any $\tau \ge \tau ^\star$ because the system remains at the isolation incapable phase once it enters the phase. Therefore from these arguments, we have $v(t)> 0$ for any $\tau \ge 0$ about the system (5) with $v(0) = v_0> 0$. Thus, we have $\hbox {d}q/\hbox {d}\tau> 0$ with $q = 0$ for any $\tau \ge 0$. Since $\hbox {d}q/\hbox {d}\tau> 0$ at $\tau = 0$, we finally find that $q\in (0, q_{\textrm{max}})$ for any $\tau> 0$.

With the same arguments, we can prove that $u> 0$ and $w> 0$ for any $\tau> 0$. Finally, since it holds from the equations of (5) that $u+v+q+w = 1$ for any $\tau \ge 0$, we obtain the lemma.

## Appendix B: Derivation of conserved quantities

*At the isolation effective phase**:*This is the phase when the system (5) follows the isolation effective phase with $\phi (q,v)=\gamma v$. First, from the equations of $\hbox {d}u/\hbox {d}\tau$ and $\hbox {d}q/\hbox {d}\tau$ in (5), we can derive the following differential equation:

B1 $$\begin{aligned} \dfrac{du}{dq}=-\dfrac{{\mathscr {R}}_0}{\gamma }\,\dfrac{u}{1- q}. \end{aligned}$$

We can easily solve equation (B1), and find equation (6) between *u* and *q*, making use of $u(0)=u_0> 0$, and $q(0)=0$.

Next, from the equations of $\hbox {d}u/\hbox {d}\tau$ and $\hbox {d}v/\hbox {d}\tau$ in (5), we can derive the following differential equation:

B2 $$\begin{aligned} \dfrac{dv}{du}= \varepsilon \dfrac{\, v\, }{u}-\varepsilon \Big (1-\dfrac{1}{\varepsilon {\mathscr {R}}_0}\Big )\dfrac{1-q}{u}-(1-\varepsilon ), \end{aligned}$$

using the relation $w=1-u-v-q$. Then substituting (6) for (B2), we can solve it and derive equation (7), making use of $u(0)=u_0$ and $v(0)=v_0 = 1-u_0$.

*At the isolation incapable phase*: Once the isolation reaches the capacity at finite time, the system (5) switches to the isolation incapable phase with $\phi (q,v)=0$. From the equations of $\hbox {d}u/\hbox {d}\tau$ and $\hbox {d}v/\hbox {d}\tau$ in (5) at the isolation incapable phase, we can obtain the following differential equation:

B3 $$\begin{aligned} \dfrac{dv}{du}= \varepsilon \dfrac{\, v\, }{u}-\varepsilon \Big (1-\dfrac{1-\gamma }{\varepsilon {\mathscr {R}}_0}\Big )\dfrac{1-q_{\textrm{max}}}{u}-(1-\varepsilon ). \end{aligned}$$

We can easily solve (B3) and get the following equation

B4 $$\begin{aligned} u(\tau )+ v(\tau )= \Big (1-\dfrac{1-\gamma }{\varepsilon {\mathscr {R}}_0}\Big )(1-q_{\textrm{max}})+C \{u(\tau )\}^\varepsilon \end{aligned}$$

from the general solution of (B3) with an undetermined constant *C*.

Since the isolation incapable phase arises only after the isolation reaches the capacity, suppose now that it arises at $\tau =\tau ^\star> 0$. From the continuity of the temporal variation of the variables in the system (5), both of equations (7) and (B4) are satisfied at $\tau =\tau ^\star$. This is the continuity condition that is satisfied by the system (5) if it switches the isolation effective phase to the isolation incapable phase at $\tau =\tau ^\star$.

First, as shown in Lemma 4.1, we find the susceptible subpopulation size $u = u^\star$ defined by (10) at the moment $\tau = \tau ^\star$ from (6), because $q(\tau ^\star ) = q_{\textrm{max}}$ at the moment when the isolation reaches the capacity. Next, from the continuity condition about equations (7) and (B4), we have the following equality which holds at $\tau =\tau ^\star$:

$$\begin{aligned}&\dfrac{1-\varepsilon {\mathscr {R}}_0 }{\gamma -\varepsilon {\mathscr {R}}_0} \Big (\dfrac{\, u^\star }{\, u_0}\Big )^{\gamma / {\mathscr {R}}_0} -\dfrac{1-\gamma }{\gamma -\varepsilon {\mathscr {R}}_0} \Big (\dfrac{\, u^\star }{\, u_0}\Big )^{\varepsilon } \\&\quad = \Big (1-\dfrac{1-\gamma }{\varepsilon {\mathscr {R}}_0}\Big )(1-q_{\textrm{max}})+C (u^\star )^\varepsilon \end{aligned}$$

when $$\gamma \ne \varepsilon {\mathscr {R}}_0$$, and

$$\begin{aligned}&\Big [\Big (\dfrac{1}{{\mathscr {R}}_0}-\epsilon \Big )\ln \dfrac{\, u^\star }{\, u_0} +1\Big ]\Big (\dfrac{\, u^\star }{\, u_0}\Big )^{\varepsilon } \\&= \Big (1-\dfrac{1-\gamma }{\varepsilon {\mathscr {R}}_0}\Big )(1-q_{\textrm{max}})+C (u^\star )^\varepsilon \\&= \Big (2-\dfrac{\, 1\, }{\gamma }\Big )(1-q_{\textrm{max}})+C (u^\star )^\varepsilon \end{aligned}$$

when $\gamma = \varepsilon {\mathscr {R}}_0$. Hence with (10), we find

B5 $$\begin{aligned} C= \left\{ \begin{array}{ll} \dfrac{1-\gamma }{\varepsilon {\mathscr {R}}_0(1-\varepsilon {\mathscr {R}}_0/\gamma )} \Big [(1-q_{\textrm{max}})^{1-\varepsilon {\mathscr {R}}_0/\gamma }-\dfrac{\varepsilon {\mathscr {R}}_0}{\gamma }\Big ](u_0)^{-\varepsilon } & \text{ when }\ \varepsilon {\mathscr {R}}_0\ne \gamma ; \\ \dfrac{1-\gamma }{\gamma } \big [ \ln (1-q_{\textrm{max}})+1 \big ](u_0)^{-\varepsilon } & \text{ when }\ \varepsilon {\mathscr {R}}_0 = \gamma . \end{array} \right. \end{aligned}$$

Finally, substituting (B5) for (B4), we can derive equation (8) for the isolation incapable phase.

## Appendix C: Proof for Lemmas 5.1, 5.2, Theorem 5.1, and Lemma 5.3

If the system remains at the isolation effective phase, the isolated subpopulation size *q* monotonically increases for any $\tau \ge 0$ since the infective subpopulation size $v> 0$ from Lemma 3.1. Since it holds that $q< q_{\textrm{max}} < 1$ for any $\tau \ge 0$, *q* must converge to a positive finite value less than or equal to $q_{\textrm{max}} < 1$ as $\tau \rightarrow \infty$, so that we have $\hbox {d}q/\hbox {d}\tau \rightarrow 0$ as $\tau \rightarrow \infty$. Therefore, it is necessary that $v\rightarrow 0$ as $\tau \rightarrow \infty$. This proves Lemma 5.1. Then, from the equation in (5), we have $\hbox {d}u/\hbox {d}\tau \rightarrow 0$ as $\tau \rightarrow \infty$ at the same time. Subsequently, from the relations $u+v+q+w=1$ and (6), we can get the disease-eliminated equilibrium $E_0^-$ given by (11), and find that $u_\infty ^-$ must be positive. This leads to Lemma 5.2.

Note that this is necessary also for the consistency with the supposition that the system always remains at the isolation effective phase. If $u\rightarrow 0$ as $\tau \rightarrow \infty$, we have $q\rightarrow 1$ as $\tau \rightarrow \infty$ because of the conserved quantity (6). This is contradictory to the supposition which means that $q< q_{\textrm{max}} < 1$ for any $\tau \ge 0$. Hence, the convergence such that $u\rightarrow u_\infty ^-> 0$ as $\tau \rightarrow \infty$ is necessary when the system always remains at the isolation effective phase.

By applying equations (6) and (7) for the equation of $\hbox {d}u/\hbox {d}\tau$ in (5), we can reduce the system (5) to the following mathematically equivalent one dimensional system:

C6 $$\begin{aligned} \dfrac{\hbox {d}u}{\hbox {d}\tau }=-{\mathscr {R}}_0u_0\{ F(u)-u\} \Big (\dfrac{u}{\, u_0}\Big )^{1-\gamma /{\mathscr {R}}_0}. \end{aligned}$$

From the above arguments to show that $\hbox {d}u/\hbox {d}\tau \rightarrow 0$ as $\tau \rightarrow \infty$ while the value *u* is positive, we find it necessary that $F(u)-u\rightarrow 0$ as $\tau \rightarrow \infty$, while $u\rightarrow u_\infty ^-$ which is given by a positive root in $(0, u_0)$ for equation (12).

Since *u* is monotonically decreasing from $u_0$ as time passes, and $F(u_0) -u_0 = 1-u_0> 0$, we have $u\rightarrow u_\infty ^-> 0$ as $\tau \rightarrow \infty$ if and only if equation (12) has a positive root $u_\infty ^-$ in $(0, u_0)$. Now the function *F*(*u*) is continuous and differentiable in $(0, u_0)$, with $F(u)\rightarrow 0$ as $u\rightarrow +0$, and $F(u) \rightarrow 1$ as $u\rightarrow u_0-0$. Moreover, from the derivative

C7 $$\begin{aligned} F'(u) = \left\{ \begin{array}{ll} \dfrac{\varepsilon }{\, u_0}\, \dfrac{1-\gamma }{\gamma -\varepsilon {\mathscr {R}}_0} \Big (\dfrac{u}{\, u_0}\Big )^{\gamma / {\mathscr {R}}_0 -1} \bigg [ \dfrac{\gamma }{1-\gamma }\Big (\dfrac{1}{\varepsilon {\mathscr {R}}_0} -1\Big ) - \Big (\dfrac{u}{\, u_0}\Big )^{\varepsilon -\gamma / {\mathscr {R}}_0} \bigg ] & \text{ when }\ \varepsilon {\mathscr {R}}_0\ne \gamma ; \\ \varepsilon \Big ( 1 + \varepsilon \dfrac{1-\gamma }{\gamma }\ln \dfrac{u}{\, u_0} + \dfrac{1-\gamma }{\gamma }\,\dfrac{1}{\, u_0} \Big ) & \text{ when }\ \varepsilon {\mathscr {R}}_0=\gamma , \end{array} \right. \end{aligned}$$

we can easily find that $F'(u)>0$ and further $F''(u)<0$ for $u\in (0,u_0)$ when $\varepsilon {\mathscr {R}}_0\ge 1$. In contrast, when $\varepsilon {\mathscr {R}}_0 < 1$, there is a unique critical value $u = u_c\in (0, u_0)$ such that $F'(u_c) = 0$, $F'(u) < 0$ for $u\in (0, u_c)$, and $F'(u)> 0$ for $u\in (u_c, u_0)$, where

C8 $$\begin{aligned} u_c := \left\{ \begin{array}{ll} u_0\Big \{ \dfrac{\gamma }{1-\gamma }\Big (\dfrac{1}{\varepsilon {\mathscr {R}}_0} -1\Big ) \Big \}^{{\mathscr {R}}_0/(\varepsilon {\mathscr {R}}_0-\gamma )} & \text{ when }\ \varepsilon {\mathscr {R}}_0\ne \gamma ; \\ u_0\exp \Big [ -\dfrac{\, 1\, }{\varepsilon }\Big (\dfrac{\gamma }{1-\gamma }+\dfrac{1}{\ u_0}\Big )\Big ] & \text{ when }\ \varepsilon {\mathscr {R}}_0= \gamma . \end{array} \right. \end{aligned}$$

From these features of the function *F*, we have $F(u)-u> 0$ for any $u\in (0, u_0)$ when and only when $\varepsilon {\mathscr {R}}_0\ge 1$. This is the case where equation (16) has no positive root in $(0, u_0)$. In contrast, there exists a unique positive root $u=u_\infty ^-$ in $(0, u_0)$ for equation (16) when and only when $\varepsilon {\mathscr {R}}_0 < 1$, proved by the intermediate value theorem for the continuous function *F*(*u*) in $[0, u_0]$, because $F(u) - u \rightarrow 0$ as $u\rightarrow +0$ and $F(u) - u\rightarrow 1-u_0>0$ as $u\rightarrow u_0-0$ while $F(u)-u < 0$ for $u\in (0, u_c)$.

Consequently, the system can always remain at the isolation effective phase only when $\varepsilon {\mathscr {R}}_0 < 1$, and then $u\rightarrow u_\infty ^-> 0$ as $\tau \rightarrow \infty$, where $u_\infty ^-$ is determined by the unique positive root in $(0, u_0)$ for equation (16). This indicates the equilibrium $E_0^{-}$ given by (17) is uniquely determined, and it is globally asymptotically stable because the temporal variation is determined by the above one dimensional ordinary differential equation (C6) when the system always remains at the isolation effective phase, about which we find from the features of *F* that $F(u) - u <0$ for $u\in (0, u_\infty ^-)$, and $F(u) - u>0$ for $u\in (u_\infty ^-, u_0)$. Lastly, these arguments prove Theorem 5.1.

Further from the above arguments, when $\varepsilon {\mathscr {R}}_0 \ge 1$, the susceptible subpopulation size *u* is monotonically decreasing toward zero, and then the isolated subpopulation size *q* monotonically increasing toward one, which is impossible unless there is no capacity for the isolation, that is, $q_{\textrm{max}} = 1$. Hence, we can conclude that *q* reaches $q_{\textrm{max}} < 1$ when $\varepsilon {\mathscr {R}}_0 \ge 1$. This proves Lemma 5.3.

## Appendix D: Proof for Theorem 6.1, Corollaries 6.1.1, 6.1.2, and Lemma 6.1

Since we have the result of Lemma 5.3 for the case of $\varepsilon {\mathscr {R}}_0\ge 1$, it is sufficient to consider only the case of $\varepsilon {\mathscr {R}}_0< 1$. As shown in (11) by the conserved quantity (6), we have equation

D9 $$\begin{aligned} q_{\infty }^{-}=1-\Big (\dfrac{u_{\infty }^{-}}{u_0}\Big )^{\gamma / {\mathscr {R}}_0} \end{aligned}$$

for the equilibrium values $u_{\infty }^{-}$ and $q_{\infty }^{-}$ at the equilibrium $E_0^-$ if the system always remains at the isolation effective phase. Then, it must be satisfied that $q_{\infty }^{-}\le q_{\textrm{max}}$. Since $q(\tau )$ is monotonically increasing in terms of $\tau$, if $q_{\infty }^{-}> q_{\textrm{max}}$, it means that the system cannot remain at the isolation effective phase, and the isolation reaches the capacity at finite time. Inversely, in a mathematical sense, when the isolation reaches the capacity at finite time, it never holds that $q_{\infty }^{-}\le q_{\textrm{max}}$, and instead it holds that $q_{\infty }^{-}> q_{\textrm{max}}$. From (D9), the condition that $q_{\infty }^{-}> q_{\textrm{max}}$ is mathematically equivalent to

D10 $$\begin{aligned} u_{\infty }^{-}<u_0(1-q_{\textrm{max}})^{{\mathscr {R}}_0/\gamma }. \end{aligned}$$

This can be regarded as the necessary and sufficient condition that the isolation reaches the capacity at finite time. As shown in Appendix C to prove Theorem 5.1 and the related results, we have found that $F(u) - u <0$ for $u\in (0, u_\infty ^-)$, and $F(u) - u>0$ for $u\in (u_\infty ^-, u_0)$ when $\varepsilon {\mathscr {R}}_0< 1$. Hence, the condition (D10) becomes equivalent to (13) of Theorem 6.1. Further, the condition (13) cannot hold if the right side

D11 $$\begin{aligned} F\big ( u_0 (1-q_{\textrm{max}} )^{{\mathscr {R}}_0/\gamma } \big ) = \left\{ \begin{array}{ll} \dfrac{1-\gamma }{\gamma -\varepsilon {\mathscr {R}}_0}(1-q_{\textrm{max}} ) \Big [\ \dfrac{1-\varepsilon {\mathscr {R}}_0 }{1-\gamma } -(1-q_{\textrm{max}} )^{\varepsilon {\mathscr {R}}_0/\gamma - 1} \Big ] & \text{ when }\ \varepsilon {\mathscr {R}}_0\ne \gamma ; \\ (1-q_{\textrm{max}} ) \Big [\ 1+\dfrac{1-\gamma }{\gamma }\ln (1-q_{\textrm{max}} ) \Big ] & \text{ when }\ \varepsilon {\mathscr {R}}_0= \gamma \end{array} \right. \end{aligned}$$

is not positive, which leads to (15) in Corollary 6.1.2.

The critical value $q_c$ for the isolation capacity $q_{\textrm{max}}$ must be defined as the upper bound of $q_{\textrm{max}}$ that satisfies the condition (D10). Therefore, it must hold that $u_{\infty }^{-}=u_0(1-q_{c})^{{\mathscr {R}}_0/\gamma }$. This proves Lemma 6.1. Then, this is the case that $u = u_0(1-q_{c})^{{\mathscr {R}}_0/\gamma }$ becomes the unique positive roof of the equation $u= F(u)$ when $\varepsilon {\mathscr {R}}_0< 1$, as the definition of $u_{\infty }^{-}$. This result shows Corollary 6.1.1.

## Appendix E: Proof for Theorem 7.1

From the equation of $\hbox {d}v/\hbox {d}\tau$ in the system (5) before and after the isolation reaches the capacity at $\tau =\tau ^\star$, we can obtain the value of $\hbox {d}v/\hbox {d}\tau$ at $q=q_{\textrm{max}}$, respectively, as follows:

E12 $$\begin{aligned} \begin{aligned}&\dfrac{\hbox {d}v}{\hbox {d}\tau }\Big |_{\tau \rightarrow \tau ^{\star }-0} = \dfrac{{\mathscr {R}}_0v^\star }{1-q_{\textrm{max}}}\Big \{u^\star -\varepsilon F(u^{\star })+\dfrac{\varepsilon {\mathscr {R}}_0-1}{{\mathscr {R}}_0} (1-q_{\textrm{max}})\Big \}; \\&\dfrac{\hbox {d}v}{\hbox {d}\tau }\Big |_{\tau \rightarrow \tau ^{\star }+0} = \dfrac{{\mathscr {R}}_0v^\star }{1-q_{\textrm{max}}}\Big \{u^{\star }-\varepsilon G(u^{\star })+\dfrac{\varepsilon {\mathscr {R}}_0-(1-\gamma )}{{\mathscr {R}}_0} (1-q_{\textrm{max}})\Big \}, \end{aligned} \end{aligned}$$

where $u(\tau ^\star ) = u^\star$ defined by (10), and we used

$$\begin{aligned} \lim _{\tau \rightarrow \tau ^\star -0}v(\tau ) = v^\star := F(u^\star )-u^\star ; \quad \lim _{\tau \rightarrow \tau ^\star +0}v(\tau ) = G(u^\star )-u^\star \end{aligned}$$

from the continuity of $u(\tau )$ and $v(\tau )$ at $\tau = \tau ^\star$. Besides, as already mentioned at the end of Sect. 4, from the continuity of $u(\tau )$ and $v(\tau )$ at $\tau = \tau ^\star$, it holds that $F(u^\star ) = G(u^\star )$.

Note that the former of (E12) is necessarily less than the latter because

$$\begin{aligned} \lim _{\tau \rightarrow \tau ^\star -0}\phi (q, v)= \gamma v^\star> \lim _{\tau \rightarrow \tau ^\star +0}\phi (q, v) = 0. \end{aligned}$$

Hence, the value *v* may continuously increase or decrease at $\tau = \tau ^\star$ unless the revival does not occur. If the former is negative and the latter is positive, it occurs. Thus, these arguments result in Theorem 7.1.

## Appendix F: Proof for Lemma 8.1 and Theorem 8.1

Applying equation (8) for the equation of $\hbox {d}u/\hbox {d}\tau$ in (5), we can obtain the following one dimensional ordinary equation which determines the dynamics by (5) for $\tau \ge \tau ^\star$ at the isolation incapable phase:

F13 $$\begin{aligned} \dfrac{\hbox {d} u }{\hbox {d}\tau } = -\dfrac{ {\mathscr {R}}_0}{1- q_{\textrm{max}} }\{ G(u) - u\} u. \end{aligned}$$

Let us consider the case of $\varepsilon {\mathscr {R}}_0 < 1$ first. Then, from the condition (ii) of Theorem 6.1, it holds that

F14 $$\begin{aligned} G(u^{\star })>u_0 (1-q_{\textrm{max}} )^{{\mathscr {R}}_0/\gamma }=u^\star =u(\tau ^{\star }), \end{aligned}$$

since $G(u^*) = F(u^*)$ as already used in Appendix E for the proof for Theorem 7.1. Thus, we have $\hbox {d}u/\hbox {d}\tau < 0$ at $\tau = \tau ^\star$ with $G(u^\star ) - u^\star> 0$. Now we consider the function $G(u)-u$ with $B\ne 0$ in order to investigate the sign of $\hbox {d}u/\hbox {d}\tau$ in $(0, u^\star )$. From the definition of *G* and *B* by (8) and (9), we have

F15 $$\begin{aligned} G(0) = \Big (1-\dfrac{1-\gamma }{\varepsilon {\mathscr {R}}_0}\Big )(1-q_{\textrm{max}}) \end{aligned}$$

and

F16 $$\begin{aligned} &G'(u) -1= \varepsilon B \Big (\dfrac{u}{\, u_0}\Big )^{\varepsilon - 1}-1 ;\\ & G''(u)= -\varepsilon (1-\varepsilon ) B \Big (\dfrac{u}{\, u_0}\Big )^{\varepsilon - 2}. \end{aligned}$$

If $B < 0$, then the function $G(u) - u$ is convex and monotonically decreasing for $u\in (0, u^\star )$ since $G''(u)> 0$ and $G'(u) - 1 < 0$ from (F16) in this case. Hence, there is no positive root of the equation $G(u)-u = 0$ if $B< 0$, because $G(u) - u> 0$ for all $u\in (0, u^\star )$ with $G(u^\star ) -u^\star> 0$.

If $B> 0$, then the function $G(u) - u$ is concave for all $u> 0$ and has at most one extremal maximum value in $(0, u^\star )$ since $G''(u) < 0$, and the number of positive root for the equation $G'(u)-1 = 0$ is only one from (F16). The function $G(u) - u$ is unimodal in $(0, u^\star )$ if the extremal maximal value exists there, while it is monotonically increasing in $(0, u^\star )$ if the extremal maximal value exists out of $(0, u^\star )$. Thus, independently of whether the function $G(u) - u$ is unimodal or monotonically increasing, there is no positive root of the equation $G(u)-u = 0$ if $G(0) \ge 0$, because then $G(u) - u> 0$ for all $u\in (0, u^\star )$ with $G(u^\star ) -u^\star> 0$. In contrast, if $G(0) < 0$, there is a unique positive root of the equation $G(u)-u = 0$ in $(0, u^\star )$, that is $u_\infty ^+$, independently of whether the function $G(u) - u$ is unimodal or monotonically increasing. Then, it holds that $G(u) - u> 0$ for $u\in (u_\infty ^+, u^\star )$ and $G(u) - u < 0$ for $u\in (0, u_\infty ^+)$. Therefore, from the temporally continuous decreasing change of *u*, we can conclude that *u* must converge to $u_\infty ^+\in (0, u^\star )$ as $\tau \rightarrow \infty$ if $B>0$ and $G(0) < 0$, while it must converge to 0 if $B< 0$ or if $B>0$ and $G(0)\ge 0$.

When $B = 0$, *G*(*u*) becomes constant for all $u\in [0, u^\star ]$:

F17 $$\begin{aligned} G(u) \equiv G(0)&=\Big (1-\dfrac{1-\gamma }{\varepsilon {\mathscr {R}}_0}\Big )(1-q_{\textrm{max}}) \nonumber \\&= \Big (1-\dfrac{1-\gamma }{\varepsilon {\mathscr {R}}_0}\Big ) \Big (\dfrac{\varepsilon {\mathscr {R}}_0}{\gamma }\Big )^{\gamma /(\gamma -\varepsilon {\mathscr {R}}_0 )} \end{aligned}$$

from (8) and (9). Then, the condition (F14) results in

$$\begin{aligned} \Big (1-\dfrac{1-\gamma }{\varepsilon {\mathscr {R}}_0}\Big ) \Big (\dfrac{\varepsilon {\mathscr {R}}_0}{\gamma }\Big )^{(\gamma -{\mathscr {R}}_0)/(\gamma -\varepsilon {\mathscr {R}}_0 )}> u_0. \end{aligned}$$

Hence, under the condition (F14) with which the system enters the isolation incapable phase, we find it necessary that $\varepsilon {\mathscr {R}}_0> 1-\gamma$. Thus, we find that $G(u) \equiv G(0) \in (0, 1)$ in this case. Since *u* is temporally monotonically decreasing from $u = u^\star$ with $G(u) \equiv G(0)>u^\star$, we have $G(u) - u = G(0) -u>0$ for any $\tau>\tau ^\star$ and any $u\in (0, u^\star )$. Therefore, *u* must converge to 0 as $\tau \rightarrow \infty$ in this case because $\hbox {d}u/\hbox {d}\tau < 0$ for any $u\in (0, u^\star )$. Finally, we can conclude that, when $\varepsilon {\mathscr {R}}_0 < 1$, *u* converges to $u_\infty ^+\in (0, u^\star )$ as $\tau \rightarrow \infty$ if and only if $B>0$ and $G(0) < 0$, and otherwise it converges to 0.

Next let us consider the case of $\varepsilon {\mathscr {R}}_0 \ge 1$. Then, we have $G(0)> 0$ from (F15). Further we necessarily have $G(u^\star ) = u^\star +v^\star> u^\star$ in this case, because $v(\tau ^\star ) = v^\star> 0$ at $\tau =\tau ^\star$ from Lemma 3.1. Thus, we can apply the same arguments as those for the case of $\varepsilon {\mathscr {R}}_0 < 1$, and find that *u* converges to 0 as $\tau \rightarrow \infty$ in this case. Consequently, we have the following result:

### Lemma F.1

At the isolation incapable phase, $u\rightarrow u_\infty ^+\in (0, u^\star )$ as $\tau \rightarrow \infty$ if and only if $\varepsilon {\mathscr {R}}_0 < 1$, $B>0$ and $G(0) < 0$. Otherwise, $u\rightarrow 0$ as $\tau \rightarrow \infty$.

From the condition $G(0) < 0$, we have $\varepsilon {\mathscr {R}}_0 < 1-\gamma$. From the condition $B>0$, we have

F18 $$\begin{aligned} \left\{ \begin{array}{ll} \Big (1-\dfrac{\varepsilon {\mathscr {R}}_0}{\gamma }\Big ) \Big [(1-q_{\textrm{max}})^{1-\varepsilon {\mathscr {R}}_0/\gamma }-\dfrac{\varepsilon {\mathscr {R}}_0}{\gamma }\Big ]>0 & \text{ when }\ \varepsilon {\mathscr {R}}_0\ne \gamma ; \\ \ln (1-q_{\textrm{max}})+1> 0 & \text{ when }\ \varepsilon {\mathscr {R}}_0= \gamma . \end{array} \right. \end{aligned}$$

When $\varepsilon {\mathscr {R}}_0 < 1-\gamma$ and $\varepsilon {\mathscr {R}}_0= \gamma$, the condition $\gamma < 1/2$ must be satisfied. In this case, the condition (ii) of Theorem 6.1 can be written as

$$\begin{aligned} 1+\ln (1-q_{\textrm{max}})> u_0 (1-q_{\textrm{max}})^{1/\varepsilon - 1}-\dfrac{1-2\gamma }{\gamma }\ln (1-q_{\textrm{max}}), \end{aligned}$$

and we find that the right side of this inequality is necessarily positive. Thus, when $\varepsilon {\mathscr {R}}_0 < 1-\gamma$ and $\varepsilon {\mathscr {R}}_0= \gamma$, the condition (F18) for $B> 0$ holds at the isolation incapable phase. Therefore, the condition $\varepsilon {\mathscr {R}}_0 < 1-\gamma$ is necessary and sufficient to have $u\rightarrow u_\infty ^+\in (0, u^\star )$ as $\tau \rightarrow \infty$ when $\varepsilon {\mathscr {R}}_0= \gamma$.

When $\varepsilon {\mathscr {R}}_0 < 1-\gamma$ and $\varepsilon {\mathscr {R}}_0\ne \gamma$, the condition (F18) becomes equivalent to the following:

F19 $$\begin{aligned} (1-q_{\textrm{max}})^{1-\varepsilon {\mathscr {R}}_0/\gamma }> \dfrac{\varepsilon {\mathscr {R}}_0}{\gamma } \quad \text{ with }\ \dfrac{\varepsilon {\mathscr {R}}_0}{\gamma } < 1\phantom {.} \end{aligned}$$

or

F20 $$\begin{aligned} (1-q_{\textrm{max}})^{1-\varepsilon {\mathscr {R}}_0/\gamma } < \dfrac{\varepsilon {\mathscr {R}}_0}{\gamma } \quad \text{ with }\ \dfrac{\varepsilon {\mathscr {R}}_0}{\gamma }> 1. \end{aligned}$$

Now, from Corollary 6.1.2, it is necessary in order to have the isolation incapable phase that the condition (15) is unsatisfied, which we can find equivalent to the following:

F21 $$\begin{aligned} (1-q_{\textrm{max}})^{1-\varepsilon {\mathscr {R}}_0/\gamma }> \dfrac{1-\gamma }{1-\varepsilon {\mathscr {R}}_0}&\quad \text{ for }\ \dfrac{\varepsilon {\mathscr {R}}_0}{\gamma } < 1; \end{aligned}$$

F22 $$\begin{aligned} (1-q_{\textrm{max}})^{1-\varepsilon {\mathscr {R}}_0/\gamma } < \dfrac{1-\gamma }{1-\varepsilon {\mathscr {R}}_0}&\quad \text{ for }\ \dfrac{\varepsilon {\mathscr {R}}_0}{\gamma }> 1. \end{aligned}$$

On the other hand, we have

$$\begin{aligned} \dfrac{1-\gamma }{1-\varepsilon {\mathscr {R}}_0} - \dfrac{\varepsilon {\mathscr {R}}_0}{\gamma } = \dfrac{(1-\gamma )-\varepsilon {\mathscr {R}}_0}{1-\varepsilon {\mathscr {R}}_0} \Big ( 1-\dfrac{\varepsilon {\mathscr {R}}_0}{\gamma } \Big ). \end{aligned}$$

Hence, when $\varepsilon {\mathscr {R}}_0 < 1-\gamma$, we find that the conditions (F21) and (F22) are sufficient for (F19) and (F20), respectively. That is, since the condition (F19) or (F20) necessarily holds when (F21) or (F22) is satisfied with $\varepsilon {\mathscr {R}}_0 < 1-\gamma$, the condition (F19) or (F20) is satisfied at the isolation incapable phase with $\varepsilon {\mathscr {R}}_0 < 1-\gamma$. Therefore, when $\varepsilon {\mathscr {R}}_0\ne \gamma$, the condition $\varepsilon {\mathscr {R}}_0 < 1-\gamma$ is necessary and sufficient to have $u\rightarrow u_\infty ^+\in (0, u^\star )$ as $\tau \rightarrow \infty$. Finally, this result and Lemma F.1 prove Lemma 8.1.

Then, from the conserved quantity (8), we note that $v\rightarrow 0$ when $u\rightarrow u_\infty ^+\in (0, u^\star )$ as $\tau \rightarrow \infty$, while $v\rightarrow G(0)> 0$ when $u\rightarrow 0$ as $\tau \rightarrow \infty$. Thus, the system (5) approaches a disease-eliminated equilibrium at the isolation incapable phase if $u\rightarrow u_\infty ^+\in (0, u^\star )$ as $\tau \rightarrow \infty$. Besides, exceptionally with $\varepsilon {\mathscr {R}}_0 =1-\gamma$, the disease goes extinct even when $u\rightarrow 0$ as $\tau \rightarrow \infty$ at the isolation incapable phase. In contrast, the system approaches the endemic equilibrium if $u\rightarrow 0$ as $\tau \rightarrow \infty$ when $\varepsilon {\mathscr {R}}_0>1-\gamma$. These results with Lemma 8.1 prove Theorem 8.1.

## Appendix G: Proof for Lemma 10.1

Taking into account the continuity of $u_\infty ^+$ in terms of $q_{\textrm{max}}\in (0,q_{c})$ when $\varepsilon {\mathscr {R}}_0 < 1-\gamma$, we prove first its monotonicity:

### Lemma G.1

$z_\infty ^+$ is monotone in terms of $q_{\textrm{max}}\in (0,q_{c})$.

### Proof

Suppose that ${\partial {z_\infty ^+}}/{\partial {q_{\textrm{max}}}} = -{\partial {u_\infty ^+}}/{\partial {q_{\textrm{max}}}}$ becomes zero for $q_{\textrm{max}}=q^\diamond \in (0,q_{c})$. First let us consider the case with $\varepsilon {\mathscr {R}}_0\ne \gamma$. From the $q_{\textrm{max}}$-derivative of equation (16) with (8) and (9), we can find that such $q^\diamond$ must satisfy the following equation:

$$\begin{aligned} -\dfrac{1-\gamma -\varepsilon {\mathscr {R}}_0}{\varepsilon {\mathscr {R}}_0} + \dfrac{1-\gamma }{\varepsilon {\mathscr {R}}_0}(1-q^\diamond )^{-\varepsilon {\mathscr {R}}_0/\gamma } \left( \dfrac{u_\infty ^+}{u_0}\right) ^\varepsilon _{q_{\textrm{max}}=q^\diamond } =0, \end{aligned}$$

that is,

$$\begin{aligned} \left( \dfrac{u_\infty ^+}{u_0}\right) ^\varepsilon _{q_{\textrm{max}}=q^\diamond } = \dfrac{1-\gamma -\varepsilon {\mathscr {R}}_0}{1-\gamma }(1-q^\diamond )^{\varepsilon {\mathscr {R}}_0/\gamma }. \end{aligned}$$

Then from (8) and (9) again, we have

$$\begin{aligned} G(u_\infty ^+)\big |_{q_{\textrm{max}}=q^\diamond } = \dfrac{1-\gamma -\varepsilon {\mathscr {R}}_0}{\gamma (1-\varepsilon {\mathscr {R}}_0/\gamma )}(1-q^\diamond ) \big [1-(1-q^\diamond )^{\varepsilon {\mathscr {R}}_0/\gamma -1}\big ] <0. \end{aligned}$$

This is contradictory to the existence of $u_\infty ^+> 0$ that satisfies equation (16) for each $q_{\textrm{max}}\in (0,q_{c})$ as shown by Lemma 8.1. Therefore, such $q^\diamond$ cannot exist in $(0,q_{c})$. Thus, the derivative ${\partial {z_\infty ^+}}/{\partial {q_{\textrm{max}}}}$ has a constant sign for $q_{\textrm{max}}\in (0,q_{c})$.

For the case with $\varepsilon {\mathscr {R}}_0= \gamma$, we can apply the same arguments to have

$$\begin{aligned} G(u_\infty ^+)\big |_{q_{\textrm{max}}=q^\diamond } = \dfrac{1-2\gamma }{\gamma }\ln (1-q^\diamond ) <0 \end{aligned}$$

since $2\gamma < 1$ when $\varepsilon {\mathscr {R}}_0= \gamma < 1-\gamma$. Hence from the contradiction again, we find that the derivative ${\partial {z_\infty ^+}}/{\partial {q_{\textrm{max}}}}$ has a constant sign for $q_{\textrm{max}}\in (0,q_{c})$ also in this case. Lastly, these arguments prove the lemma. $\square$

Next to complete the proof for Lemma 10.1, we prove the following feature of the derivative ${\partial {z_\infty ^+}}/{\partial {q_{\textrm{max}}}}$:

### Lemma G.2

$$\left. \dfrac{\partial {z_\infty ^+}}{\partial {q_{\textrm{max}}}}\right| _{q_{\textrm{max}}\rightarrow 0+}<0$$.

### Proof

As $q_{\textrm{max}}\rightarrow 0+$, equation (16) becomes

G23 $$\begin{aligned} 1-u_\infty ^+ = \dfrac{1-\gamma }{\varepsilon {\mathscr {R}}_0}\Big [1-\Big (\dfrac{u_\infty ^+}{\, u_0}\Big )^{\varepsilon }\Big ] \end{aligned}$$

for both cases of $\varepsilon {\mathscr {R}}_0\ne \gamma$ and $\varepsilon {\mathscr {R}}_0= \gamma$. It is easily found that this equation has a unique positive root $u_\infty ^+ = u_\infty ^{+0}\in (0, u_0)$. From equation (16) with (8) and (9), we can derive

G24 $$\begin{aligned} \left. \dfrac{\partial {z_\infty ^+}}{\partial {q_{\textrm{max}}}}\right| _{q_{\textrm{max}}\rightarrow 0+} = -\left. \dfrac{\partial {u_\infty ^+}}{\partial {q_{\textrm{max}}}}\right| _{q_{\textrm{max}}\rightarrow 0+} = \dfrac{u_\infty ^{+0}}{1-G'(u_\infty ^{+0})}, \end{aligned}$$

making use of (G23).

First let us consider the case with $\varepsilon {\mathscr {R}}_0\ne \gamma$. Then from (8) and (9) with (G23) again, we have

G25 $$\begin{aligned} 1-G'(u_\infty ^{+0})&= 1-\dfrac{\,\varepsilon \,}{\ u_0}\Big (\dfrac{u_\infty ^{+0}}{u_0}\Big )^{\varepsilon -1}\Big (\dfrac{1-\gamma }{\varepsilon {\mathscr {R}}_0}\Big ) \nonumber \\&= 1-\dfrac{\,\varepsilon \,}{\ u_\infty ^{+0}}\Big (\dfrac{u_\infty ^{+0}}{u_0}\Big )^{\varepsilon }\Big (\dfrac{1-\gamma }{\varepsilon {\mathscr {R}}_0}\Big ) \nonumber \\&= \dfrac{1-u_\infty ^{+0}}{u_\infty ^{+0}} \left[ \ \dfrac{u_\infty ^{+0}}{1-u_\infty ^{+0}} - \varepsilon \dfrac{(u_\infty ^{+0}/u_0)^\varepsilon }{1-(u_\infty ^{+0}/u_0)^\varepsilon } \ \right] . \end{aligned}$$

Now consider the function $\zeta (\varepsilon ):= \varepsilon a^\varepsilon /(1-a^\varepsilon )$ for $\varepsilon \in (0, 1)$ with $a\in (0, 1)$. We can easily find that

$$\begin{aligned} \zeta '(\varepsilon ) = \dfrac{a^\varepsilon }{(1-a^\varepsilon )^2}\big (1-a^\varepsilon +\varepsilon \ln a\big ) < 0 \end{aligned}$$

because the function $h(x):= 1-x^\varepsilon +\varepsilon \ln x$ is negative for $x\in (0, 1)$ about any $\varepsilon \in (0, 1)$. Hence, the function $\zeta (\varepsilon )$ is monotonically decreasing in terms of $\varepsilon$, so that we have $\zeta (\varepsilon )> \zeta (1) = \xi (a):= a/(1-a)$. Since $\xi (a)$ is monotonically increasing in terms of $a\in (0, 1)$, we finally find the following order:

$$\begin{aligned} \zeta (\varepsilon )\big |_{a = u_\infty ^{+0}/u_0}> \zeta (1 )\big |_{a = u_\infty ^{+0}/u_0} = \xi \big ( u_\infty ^{+0}/u_0\big )> \xi \big ( u_\infty ^{+0}\big ) \end{aligned}$$

because $u_\infty ^{+0}< u_0<1$. Then, since equation (G25) can be rewritten as

$$\begin{aligned} 1-G'(u_\infty ^{+0}) = \dfrac{1}{\xi \big ( u_\infty ^{+0}\big )} \left[ \ \xi \big ( u_\infty ^{+0}\big )-\zeta (\varepsilon )\big |_{a = u_\infty ^{+0}/u_0} \ \right] , \end{aligned}$$

we conclude that $1-G'(u_\infty ^{+0}) < 0$, so that the derivative (G24) is negative. These arguments can be simply applied for the case of $\varepsilon {\mathscr {R}}_0 = \gamma$, and show that the derivative (G24) is negative. Consequently, we have proved that the derivative (G24) is negative. $\square$

From the continuity of $u_\infty ^+$ in terms of $q_{\textrm{max}}\in (0,q_{c})$, Lemmas G.1 and G.2 prove that the $q_{\textrm{max}}$-derivative of $z_\infty ^+$ is negative for $q_{\textrm{max}}\in (0,q_{c})$. As a result, Theorem 10.1 has been proven.

## Appendix H: Proof for Theorem 10.1

First we show the following lemma:

### Lemma H.1

$$z^{\dagger }_\infty \ge z_{\infty }^{-}$$.

### Proof

If the system (5) enters the isolation incapable phase at time $\tau =\tau ^{\star }$ with $q_{\textrm{max}}<q_c$, we have $u_{\infty }^{+}<u(\tau ^{\star })=u^\star$ because $\hbox {d}u/\hbox {d}\tau <0$ even after $\tau =\tau ^{\star }$. Hence, $z_{\infty }^{+}:=1-u_{\infty }^{+}>1-u^\star$ for $q_{\textrm{max}}<q_c$ with $u(\tau ^{\star })=u^\star$ given by (10). Therefore, we find that

H26 $$\begin{aligned} \lim _{q_{\textrm{max}} \rightarrow q_{c}-0} z_\infty ^+ = z_\infty ^\dagger \ge \lim _{q_{\textrm{max}} \rightarrow q_{c}-0} (1-u^\star ) = 1-u_0 (1-q_c )^{{\mathscr {R}}_0/\gamma }. \end{aligned}$$

Then from Lemma 6.1, the right side of (H26) is equal to $z_{\infty }^{-}$, which proves this lemma. $\square$

As shown in the first part of Sect. 10, if $\varepsilon {\mathscr {R}}_0\ge 1-\gamma$ and $q_{\textrm{max}}<q_{c}$, we have the final epidemic size $z_\infty = z_\infty ^{+}=1$. From Theorem 6.1 and Corollary 6.1.1, if $\varepsilon {\mathscr {R}}_0\ge 1$, we have $q_c = 1$, and thus there is no critical capacity for the isolation. Then, we do not have any case of the final epidemic size at the isolation effective phase, that is, $z_\infty ^-$ does not exist. Only if $\varepsilon {\mathscr {R}}_0< 1$, we can have $z_\infty ^-$ as the final epidemic size at the isolation effective phase. Therefore, we can conclude that, the final epidemic size $z_\infty$ shows a discontinuity at $q_{\textrm{max}} = q_c$ for $\varepsilon {\mathscr {R}}_0\in [\, 1-\gamma , 1)$, when $z_\infty ^{+}=1> z_\infty ^-$ which is given by Lemma 6.1.

If $\varepsilon {\mathscr {R}}_0< 1-\gamma$ and $q_{\textrm{max}}<q_{c}$, we have the final epidemic size $z_\infty = z_\infty ^+ < 1$ when the system (5) approaches a disease-eliminated equilibrium $E_0^{+}$ given by (17). Then, the final epidemic size $z_\infty ^+:=1-u_\infty ^+$ is determined by the unique positive root $u^{+}_\infty$ of equation (16) in Lemma 8.1. As argued in Appendix F for the proof of Lemma 8.1, the unique existence of the positive root $u^{+}_\infty$ of equation (16) follows the condition that $G(u^\star )-u^\star> 0$ and $G(0) < 0$, where the function $G(u) - u$ with $q_{\textrm{max}} \in (0, q_c)$ is unimodal or monotonically increasing in $(0, u^\star )$.

Now we can find that

### Lemma H.2

$u^\star \rightarrow u_\infty ^-$, $G(u^\star )-u^\star \rightarrow G(u_\infty ^-)- u_\infty ^-= 0$, and $G(0) < 0$ as $q_{\textrm{max}} \rightarrow q_c-0$ with $\varepsilon {\mathscr {R}}_0 <1-\gamma$.

This lemma can be easily proved by the straightforward calculation with (7–9), (10), (14), and Lemma 6.1. Hence from the continuity of $u_\infty ^+$ in terms of $q_{\textrm{max}}\in (0, q_c)$, we have $u_\infty ^+\rightarrow u_\infty ^-$ as $q_{\textrm{max}} \rightarrow q_c-0$ if the function $G(u) - u$ becomes monotonically increasing in $(0, u^\star )$ as $q_{\textrm{max}} \rightarrow q_c-0$. This is because there is no root of the equation $G(u) - u =0$ in $(0, u^\star )$ as $q_{\textrm{max}} \rightarrow q_c-0$, while $u_\infty ^+$ is continuous in terms of $q_{\textrm{max}}\in (0, q_c)$. In contrast, if the function $G(u) - u$ becomes unimodal with a maximal extremum in $(0, u^\star )$ as $q_{\textrm{max}} \rightarrow q_c-0$, the equation $G(u)-u = 0$ has a root $u_\infty ^{++}\in (0, u^\star )$, and we have $u_\infty ^+\rightarrow u_\infty ^{++}$ as $q_{\textrm{max}} \rightarrow q_c-0$ because of the continuity of $u_\infty ^+$ in terms of $q_{\textrm{max}}\in (0, q_c)$.

As shown in Appendix F, the continuous function $G(u) - u$ has at most one extremum for $u> 0$, and $G'(u) -1= (\varepsilon /u_0)B(u/u_0)^{\varepsilon -1}-1 \rightarrow \infty$ as $u\rightarrow +0$ with $\varepsilon {\mathscr {R}}_0 <1-\gamma$. Thus, it is monotonically increasing in $(0, u^\star )$ if and only if the derivative of $G(u)-u$, that is, $G'(u)-1$ is nonnegative for $u = u^\star$, while it is unimodal in $(0, u^\star )$ if $G'(u)-1$ is negative for $u = u^\star$. As a result, taking account of Lemma H.2 and the above arguments on the limit of $u_\infty ^+$ as $q_{\textrm{max}} \rightarrow q_c-0$, we get

### Lemma H.3

As $$q_{\textrm{max}} \rightarrow q_c-0$$ with $$\varepsilon {\mathscr {R}}_0 <1-\gamma$$,

$$\begin{aligned} u_\infty ^+ \rightarrow \left\{ \begin{array}{ll} u_\infty ^- & \ \text{ if }\ \ G'(u_\infty ^-)\ge 1; \\ u_\infty ^{++}<u_\infty ^- & \ \text{ if }\ \ G'(u_\infty ^-)< 1. \end{array} \right. \end{aligned}$$

Therefore, if and only if $G'(u_\infty ^-)< 1$ with $\varepsilon {\mathscr {R}}_0 <1-\gamma$, we have $z_\infty ^\dagger =1-u_\infty ^{++}> 1-u_\infty ^- = z_\infty ^-$. The condition $G'(u_\infty ^-)< 1$ with $\varepsilon {\mathscr {R}}_0 <1-\gamma$ becomes (19) in Theorem 10.1 by the straightforward calculation with Lemma 6.1 and (14). The calculation must be carried out, respectively, for the cases of $\varepsilon {\mathscr {R}}_0\ne \gamma$ and $\varepsilon {\mathscr {R}}_0= \gamma$, while the final result for $\varepsilon {\mathscr {R}}_0= \gamma$ appears to be included in (19).

Making use of (14) in a different way, the condition (19) can be rewritten as

H27 $$\begin{aligned}&\dfrac{1-\gamma }{\gamma -\varepsilon {\mathscr {R}}_0} (1-q_c)^{\varepsilon {\mathscr {R}}_0-\gamma } < \dfrac{1-\varepsilon {\mathscr {R}}_0}{\gamma -\varepsilon {\mathscr {R}}_0} \nonumber \\&\quad - \dfrac{\varepsilon }{1-\varepsilon }\Big (\dfrac{1-\gamma }{\varepsilon {\mathscr {R}}_0}-1\Big ) \ \ \text{ for }\ \ \varepsilon {\mathscr {R}}_0\ne \gamma ; \end{aligned}$$

H28 $$\begin{aligned}&\dfrac{1-\gamma }{\gamma }\ln (1-q_c)> \dfrac{\varepsilon }{1-\varepsilon }\dfrac{1-2\gamma }{\gamma }-1 \ \ \text{ for }\ \ \varepsilon {\mathscr {R}}_0= \gamma . \end{aligned}$$

Since $1-q_c \in (0, 1)$, it is necessary that the right side of (H27) is greater than $(1-\gamma )/(\gamma -\varepsilon {\mathscr {R}}_0)$, and the right side of (H28) is negative. Then, we can find the following necessary condition:

### Lemma H.4

For $G'(u_\infty ^-)< 1$ with $\varepsilon {\mathscr {R}}_0 <1-\gamma$, it is necessary that ${\mathscr {R}}_0> 1-\gamma$.

Finally, Lemmas H.3 and H.4 prove Theorem 10.1.
